# Performance Evaluation of Four Deep Learning-Based CAD Systems and Manual Reading for Pulmonary Nodules Detection, Volume Measurement, and Lung-RADS Classification Under Varying Radiation Doses and Reconstruction Methods

**DOI:** 10.3390/diagnostics15131623

**Published:** 2025-06-26

**Authors:** Sifan Chen, Lingqi Gao, Maolu Tan, Ke Zhang, Fajin Lv

**Affiliations:** Department of Radiology, First Affiliated Hospital of Chongqing Medical University, Chongqing 400016, China; 2023140183@stu.cqmu.edu.cn (S.C.); glq18sci@stu.cqmu.edu.cn (L.G.); 2024120663@stu.cqmu.edu.cn (M.T.); 2023110179@stu.cqmu.edu.cn (K.Z.)

**Keywords:** computer-aided diagnosis, deep learning, volume rendering, pulmonary nodule, phantom study

## Abstract

**Background:** Optimization of pulmonary nodule detection across varied imaging protocols remains challenging. We evaluated four DL-CAD systems and manual reading with volume rendering (VR) for performance under varying radiation doses and reconstruction methods. VR refers to a post-processing technique that generates 3D images by assigning opacity and color to CT voxels based on Hounsfield units. **Methods**: An anthropomorphic phantom with 169 artificial nodules was scanned at three dose levels using two kernels and three reconstruction algorithms (1080 image sets). Performance metrics included sensitivity, specificity, volume error (AVE), and Lung-RADS classification accuracy. **Results**: DL-CAD systems demonstrated high sensitivity across dose levels and reconstruction settings, with three fully automatic DL-CAD systems (0.92–0.95) outperforming manual CT readings (0.72), particularly for sub-centimeter nodules. However, DL-CAD systems exhibited limitations in volume measurement and Lung-RADS classification accuracy, especially for part-solid nodules. VR-enhanced manual reading outperformed original CT interpretation in nodule detection, particularly benefiting less-experienced radiologists under suboptimal imaging conditions. **Conclusions**: These findings underscore the potential of DL-CAD for lung cancer screening and the clinical value of VR in low-dose settings, but they highlight the need for improved classification algorithms.

## 1. Introduction

Accurate detection and characterization of pulmonary nodules remain critical for lung cancer screening, where early diagnosis significantly reduces mortality [1,2,3,4]. The increasing volume and complexity of medical imaging data from reduced-dose CT protocols highlight human cognitive limitations [5,6], manifesting clinically through diagnostic errors, prolonged interpretation times, and suboptimal workflow efficiency [7]. Artificial intelligence offers potential solutions through computer-aided diagnosis (CAD) systems [8], with numerous deep learning-based CAD (DL-CAD) systems now commercially available following regulatory approvals [9,10].

While existing literature supports CAD’s technical feasibility for nodule quantification and characterization [11,12], critical knowledge gaps persist regarding comparative system performance across heterogeneous imaging parameters and reconstruction methodologies. Current evidence remains predominantly constrained to pre-clinical CAD systems, with limited evaluation of commercially available DL-CAD implementations, which have passed the initial research and development phase and are currently available for clinical use. Critical factors affecting CAD performance include radiation dose, reconstruction kernel and algorithm, and nodule characteristics [13,14,15,16]. Furthermore, substantial performance variation exists among published CAD systems due to algorithmic heterogeneity and training data differences [17]. Therefore, it is important to test and compare the different DL-CAD systems under the diversity of influencing factors to reveal unforeseen challenges and to allow for adjustments in diagnostic workflows.

Conventional manual interpretation remains central to clinical workflows. Post-processing technique volume rendering (VR) has shown potential to improve nodule detection by enhancing nodule visibility via three-dimensional spatial visualization and noise reduction through the customized CT value-opacity settings [18,19,20].

This study, therefore, aims to (1) evaluate four commercial DL-CAD systems in detecting pulmonary nodules, measuring volumes, and classifying Lung-RADS categories under varying radiation doses, and reconstruction kernels and algorithms; (2) assess manual interpretation performance using both VR and original CT images. The secondary objective examines VR’s utility in low-dose and ultra-low-dose screening environments.

## 2. Materials and Methods

### 2.1. Chest Phantoms

**Figure 1:**
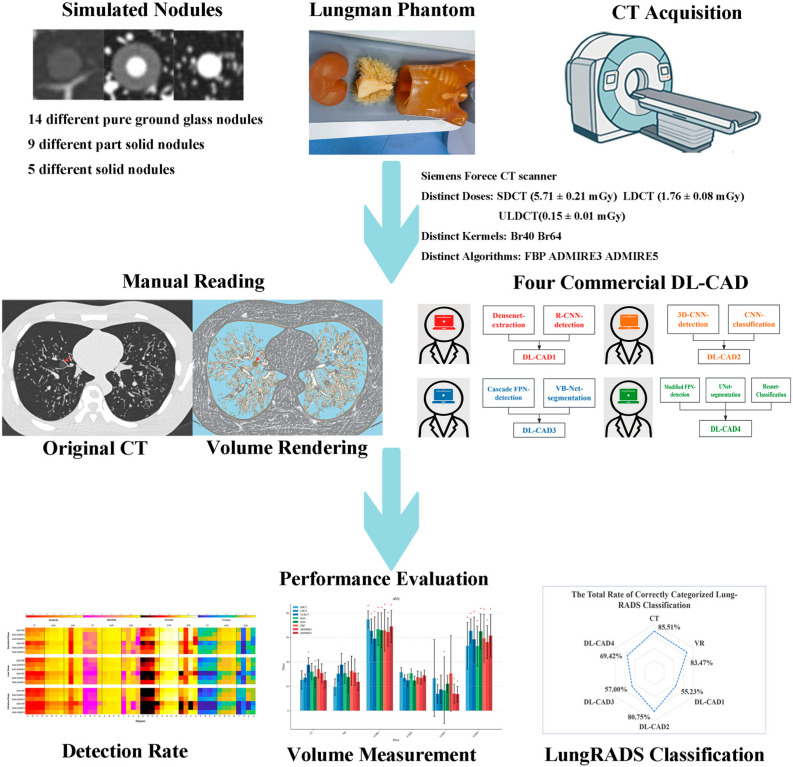
Flowchart showing the experimental process, including the phantom’s set-up, data acquisition, imaging analysis, and performance evaluation. Red arrow indicates the artificial nodule in the phantom.

An anthropomorphic thorax phantom (Multipurpose Chest Phantom N1 Lungman; Kyoto Kagaku, Kyoto, Japan) with a simulated tissue-equivalent extension layer representing the average male body weight was used in this study. The phantom was composed of a chest wall, spine, and ribs, with an integrated insert replicating cardiac, vascular, and mediastinal structures. As established in prior validation studies, this phantom demonstrates radiological properties closely approximating human tissue characteristics [21,22]. An overview of the phantom’s set-up and the research pipelines are presented in Figure 1.

In total, 28 artificial pulmonary nodules were used (Kyoto Kagaku, Kyoto, Japan), as follows: 5 artificial solid nodules (SNs) with a density of +100 Hounsfield units [HU] (3, 5, 8, 10, and 12 mm), 14 artificial ground-glass nodules (GGNs) with a density range of −800 HU to −350 HU (3, 5, 8, 10, 12, and 15 mm), and 9 artificial part-solid nodules (PSNs) with a non-solid component density of −650 HU and a solid component density of −50 HU or 0 HU (15 or 20 mm for a whole nodule and 3, 5, 7, and 9 mm for a solid component). Nodule distribution was determined using a MATLAB randomization algorithm (R2018b, The Mathworks, Inc., Natick, MA, USA) accounting for the following five key parameters: quantity (1–5 nodules per phantom), density classification, size category, lung segment, and central/peripheral location. Artificial nodules were subsequently positioned within the assigned anatomical segment between the pulmonary vessels of the Lungman phantom by an independent operator who was blinded to subsequent image analysis procedures, with immediate CT confirmation of the placement accuracy prior to experimental scanning. A total of 60 phantom arrangements were employed, containing a total of 169 artificial nodules, with the final distribution being 71 solid nodules, 59 pure ground-glass nodules, and 39 part-solid nodules. Further details of the artificial nodules are provided in Supplementary Table S1.

### 2.2. Image Acquisition and Reconstruction

All images were prospectively acquired using a third-generation dual-source CT scanner (SOMATOMA Force; Siemens Healthcare, Forchheim, Germany). The image acquisitions were performed using the following three radiation dose levels: standard-dose computed tomography (SDCT) at 120 kVp and a 100 mAs quality reference; low-dose computed tomography (LDCT) at 100 kVp and a 50 mAs quality reference; and ultra-low-dose computed tomography (ULDCT) at Sn100 kVp and a 45 mAs quality reference. The images were reconstructed using either a soft-tissue kernel (Br40) or a sharp reconstruction kernel (Br64) with a slice thickness of 1 mm. The reconstruction algorithms employed were filtered back projection (FBP) and advanced modeled iterative reconstruction (ADMIRE) at strength levels of 3 and 5. Other acquisition parameters were as follows: collimation, 128 × 0.6 mm; pitch, 1.2; gantry rotation time, 0.25 s; and image matrix, 512 × 512 pixels. Each phantom configuration yielded 18 image sets, resulting from the combination of three dose levels, two kernel types, and three reconstruction algorithms, culminating in a total of 1080 image sets and 3042 simulated lung nodules. The mean volumetric CT dose index (CTDIvol) for the three dose groups was as follows: SDCT: 5.71 ± 0.21 mGy, LDCT: 1.76 ± 0.08 mGy, and ULDCT: 0.15 ± 0.01 mGy. Detailed information on the CT protocols is summarized in Table 1.

### 2.3. Deep Learning CAD Systems

This study involved the evaluation of four commercial DL-CAD systems embodying an advanced deep neural network with specific emphasis on pulmonary nodules. The principle of all DL-CAD systems has previously been described, and all systems have been officially approved for clinical use, as follows: CAD1 (InferRead CT Lung, InferVision Medical Health, Beijing, China), CAD2 (VD20A of syngo.via VB40, Siemens Healthcare, Forchheim, Germany), CAD3 (uAI-ChestCare, United Imaging Healthcare, Shanghai, China), and CAD4 (LungDoc, Shukun Technology, Beijing, China).

Specifically, CAD1 is composed of two convolutional neural network (CNN) models, combining a DenseNet for feature extraction and a modified Faster R-CNN detector adapted for 2.5D CT scan analysis by processing successive 2D slices without 3D convolutions [23]. CAD2 employs a deep 3D CNN for lung nodule detection, performing preprocessing, candidate generation, and cascaded CNN-based classification to distinguish true nodules from false positives [24]. CAD3 is based on a deep learning model with a 3D ReLU cascade FPN for detecting tiny lung nodules and VB-Net for segmentation [25]. CAD4 utilizes a modified FPN method incorporating a multi-level feature fusion strategy and attention mechanism for the nodule detection, followed by UNet for segmentation and ResNet for classification [26]. CAD1, CAD3, and CAD4 are capable of automatic pulmonary nodule detection and provide detailed nodule characteristics, including layer, anatomical location, nodule type, longest transverse diameter and maximal perpendicular short axis, volume, solid component diameter and volume, and Lung-RADS v2022 classification. In contrast, CAD2 featured automatic nodule detection with semi-automatic measurement capabilities, allowing for subsequent manual adjustments. With CAD2, the semi-automatically measured average diameter and volume of the detected nodules were recorded by two independent radiology interns, who then derived the Lung-RADS classification. Detailed information on the involved DL-CAD systems is summarized in Table 2.

All reconstructed images were transferred from the scanner to the PACS (picture archiving and communication system), where all image sets were independently assessed by each DL-CAD system for nodule detection, volume measurement, and Lung-RADS classification. The results of the image analyses were then exported to an image processing workstation equipped with the clients of the four DL-CAD systems and a high-definition liquid-crystal display monitor. The final outcomes were subsequently reviewed by an independent radiology resident, who meticulously compared the exported results for each nodule with the ground truth (GT) data, thereby ascertaining the comparative performances of the four DL-CAD systems.

### 2.4. Image Analysis

Volume rendering (VR) is a three-dimensional post-processing technique that assigns continuous opacity values and colors to each voxel based on Hounsfield units (HU), enabling visualization of tissue gradients and spatial relationships. Although maximum intensity projection (MIP) is the established post-processing standard in lung cancer screening proven to enhance detection rates of pulmonary nodule [27], VR exhibits two primary advantages critical to this study, as follows: superior measurement accuracy and enhanced tolerance to suboptimal image quality. First, VR preserves depth information and accurately depicts three-dimensional spatial relationships between lesions and surrounding tissues, whereas MIP as a flattened projection that suffers from potential structural overlap. Furthermore, VR simultaneously visualizes tissues across density gradients (e.g., ground-glass opacities alongside solid components), while MIP exclusively highlights high-density structures (e.g., vessels/calcifications), obscuring low-contrast soft-tissue details [28]. In addition, a previous study has suggested a strong correlation of VR measurement with the pathological finding [19]. Jointly, these characteristics enable VR to provide more precise volumetric measurements. Second, the established literature [29] and findings from the current study demonstrate VR’s noise-suppression capability, particularly under reduced-dose protocols, such as low-dose (LDCT) and ultra-low-dose (ULDCT) scanning (Supplementary Figure S1). Given these advantages in measurement fidelity and noise resilience, although VR is not yet adopted in screening guidelines, it was selected over MIP for integration into the manual reading workflow in the current study.

**Figure 2:**
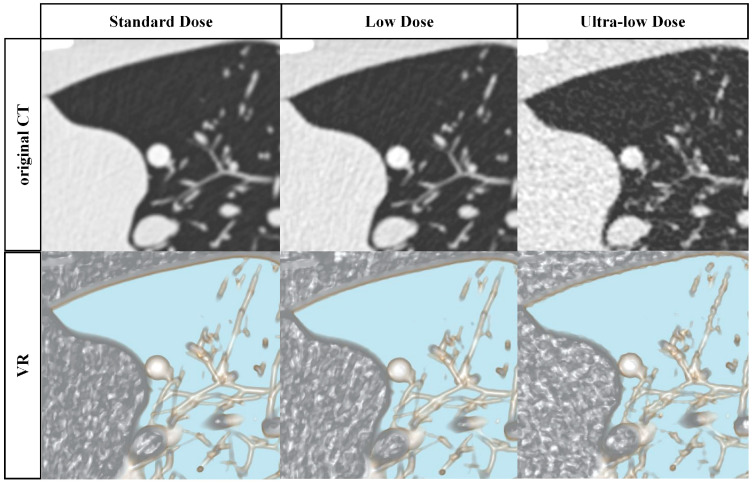
Representative chest CT images and corresponding volume rendering (VR) images from standard-dose computed tomography (SDCT), low-dose computed tomography (LDCT), and ultra-low-dose computed tomography (ULDCT), reconstructed using the Br40 kernel and ADMIRE−3 algorithm. The images reveal a solid nodule in the left upper lobe with a CT attenuation of 100 HU. The solid nodule appears clearer and more detailed in the axial CT images under SDCT and LDCT scanning compared with ULDCT. However, the nodule remains distinctly visible in the VR images, even under ULDCT scanning. Ultimately, the nodule was missed in both the axial CT images and the CAD2 analysis.

Four radiologists (a second-year radiology resident (reader A), two attending radiologists with 5 and 7 years of experience in chest imaging (readers B and C), and a board-certified radiologist with 20 years of experience (reader D)) were asked to independently review the original CT images and volume rendering (VR) on a PACS workstation (Vue PACS, Carestream, version 12.2.6.3000020), with standard lung window settings (width: 1600 Hounsfield units (HU) and level: −600 HU) for both the original CT and VR groups. For VR post-processing, all CT images were exported to the PACS clients, where the software allowed for real-time 3D processing of VR reconstruction without manual user interaction, with the whole volume of each image set being reconstructed with a slab thickness of 10 mm. The opacity-CT value curve was preset in the PACS client and is provided in Supplementary Figure S2. Fixed VR parameters were employed for manual reading, with no adjustments permitted to ensure the elimination of human-induced biases. Prior to the formal image analysis, all observers received training in PACS operation and were familiar with the morphology of pulmonary nodules on VR images. Figure 2 illustrates the comparative representation of the VR and original CT images. Initially, the observers were blinded to the characteristics of the simulated nodules. Each observer conducted separate image analyses of the original CT and VR images, with a two-month interval between analyses to reduce potential recall bias. During each viewing session, images of varying dose levels and reconstruction settings, including different kernels and algorithms, were presented in a randomized sequence. A four-week interval was set between each set of images (every 6 groups, 360 volumes). Every observer was required to record the layer, location, density type, longest transverse diameter and perpendicular short axis, and solid component diameter of each observed nodule on the axial CT and VR images, as well as the Lung-RADS classification for each nodule. Additionally, they provided a subjective image quality rating on a 5-point Likert scale, as follows: 1 indicated nondiagnostic image quality with strong artifacts, insufficient for diagnostic purposes; 2 reflected severe artifacts, with uncertainty about the evaluation; 3 signified moderate artifacts, with a restricted assessment; 4 indicated slight artifacts, allowing for an unrestricted diagnostic image evaluation; and 5 represented excellent image quality, with no artifacts. CT images scoring 3 to 5 on the Likert scale were deemed diagnostic. A pre-prepared timer app automatically recorded the time each observer spent on image reading.

### 2.5. Statistical Analysis

Statistical analyses were conducted using SPSS software (version 26.0; SPSS, Chicago, IL, USA) and the statistical program R (version 4.4.1, https://www.r-project.org/). Continuous variables are expressed as the mean ± standard deviation (SD). The evaluation of the performances by the DL-CAD systems and manual reading is based on several metrics, including sensitivity, specificity, accuracy, precision, F1 score, and absolute volume error (AVE). The calculation formulas for these metrics are provided in the Supplementary Material. The chi-square test, followed by a z-test, was employed to compare nodule detectability and matching rates of Lung-RADS classification across interpretation tools and doses. A comparison of the reading time and image-quality score among groups was performed with the Friedman test followed by the post hoc test. A comparison of the absolute volume errors (AVEs) among groups was performed with repeated measures analysis of variance, applying Greenhouse–Geisser correction followed by post hoc tests. For the nodule-level analysis, independent predictors of detection rates and matching rates of Lung-RADS classification—including nodule density (solid, ground-glass, and part-solid), diameter (≤5 mm, 5–10 mm, 10–15 mm, and 15–20 mm), location (peripheral and central), Lung-RADS classification (2, 3, 4A, and 4B), dose level (standard dose, low dose, and ultra-low dose), and reconstruction kernel (Br40 and Br64) and algorithm (FBP, ADMIRE−3, and ADMIRE−5)—were identified using multivariate binary logistic regression. Factors influencing the volumetry measurement were explored using a generalized linear mixed model with the aforementioned factors as fixed effects and subject identification number (and readers) as random effects. Multiple comparisons were corrected using the Bonferroni method, and a two-sided *p*-value less than 0.05 was considered as indicating statistical significance.

## 3. Results

### 3.1. Overall Nodule Detection Performance

Table 3 provides details on the nodule detection performances of the four DL-CAD systems and manual readings across all dose levels, and reconstruction kernels and algorithms. Three fully automatic systems (CAD1, CAD3, and CAD4) achieved high sensitivity (0.92–0.95), specificity (0.78–0.90), and accuracy (0.84–0.91), while the semi-automatic system (CAD2) demonstrated lower sensitivity (0.68) but maintained specificity (0.94) and accuracy (0.85). At standard and low-dose CT, all systems showed comparable nodule detection performances, though sensitivity reductions of 0.09, 0.11, and 0.07 were observed for CAD1, CAD3, and CAD4, respectively, at an ultra-low dose, accompanied by specificity decreases of 0.23 (CAD1) and 0.08 (CAD3) and accuracy declines of 0.18 (CAD1) and 0.09 (CAD3). In contrast, CAD2 exhibited improved sensitivity (+0.10), specificity (+0.04), and accuracy (+0.06) at reduced dose levels compared to the standard dose. In addition, nodule detection varied for all systems in the reconstruction kernel and algorithm subgroups, where superior performances were observed for the smooth kernel (Br40) and high-strength iterative reconstruction (ADMIRE−5) groups. For the manual reading, VR demonstrated significantly superior performances over the original CT in sensitivity (0.92 vs. 0.72, *p* < 0.05), specificity (0.98 vs. 0.81, *p* < 0.05), and accuracy (0.95 vs. 0.77, *p* < 0.05). VR maintained consistent performances across the dose and reconstruction subgroups, while the original CT readings showed declines in performance under ultra-low-dose settings, using the sharp kernel (Br64), and with filtered back projection (FBP) reconstruction. In terms of sensitivity, VR significantly outperformed CAD2 (*p* < 0.05). However, VR showed no statistically significant difference compared with the three automatic CAD systems across the dose, kernel, and algorithm subgroups (*p* > 0.05). While VR demonstrated significantly superior specificity and accuracy over the automatic CAD systems in specific subgroups (i.e., Br64 and FBP), the CT detection performance differed significantly from both VR and the three automatic CAD systems (*p* < 0.05) (Supplementary Table S2).

**Figure 3:**
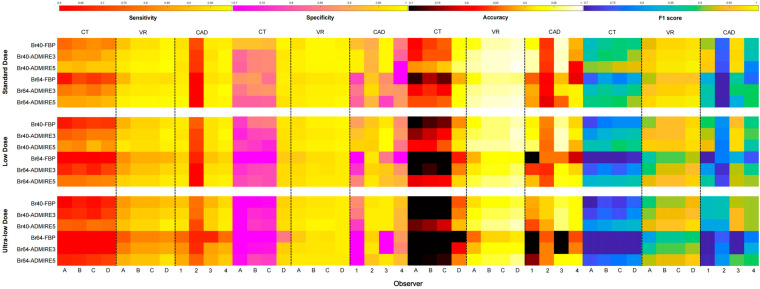
Heatmap presentation of the performances of the CT, VR, and CAD systems (1, 2, 3, and 4). The four observers’ (A, B, C, and D) sensitivity, specificity, accuracy, and F1 score are plotted for each dose, kernel, and algorithm combinations. The first column represents sensitivity, the second column shows specificity, the third column shows accuracy, and the forth column shows F1 score.

The comprehensive performance characteristics of the four DL-CAD systems and four observers using the VR and original CT readings under each dose–kernel–algorithm combination are visually summarized by a heatmap (Figure 3). A critical analysis reveals system-specific variability for the optimal dose and reconstruction parameters that achieved an optimal sensitivity–specificity balance. While most DL-CAD systems attained superior performance metrics under standard/low-dose with smooth kernel and iterative reconstruction algorithms, CAD3 and CAD4 maintained clinically acceptable performances even under ultra-low-dose protocols using sharp kernels with FBP. Manual reading assessments demonstrated substantial VR-enhanced diagnostic capabilities, with observer-specific improvements spanning sensitivity (Δ0.06–0.44), specificity (Δ0.04–0.66), accuracy (Δ0.04–0.55), and F1 scores (Δ0.05–0.43), with maximum improvements observed in the ultra-low-dose Br64-FBP subgroup. Additionally, resident radiologist using VR achieved a significantly better diagnostic sensitivity than the board-certified specialists interpreting original CT images (*p* < 0.05, Supplementary Table S3). Meanwhile, VR significantly enhanced the reading speed (*p* < 0.05) and image quality (*p* < 0.05) compared with the original CT images (Supplementary Table S5 and Figure S3).

### 3.2. Subgroup Analysis of Nodule Detection

Subgroup analyses of the nodule detection sensitivity were systematically performed across the DL-CAD systems and manual readings to evaluate performance variations under diverse nodule characteristics (Table 4). CAD1, CAD3, and CAD4 demonstrated significantly higher sensitivities than the manual readings (*p* < 0.05) for nodules smaller than 10 mm, solid/ground-glass density, and Lung-RADS category 2, which are the most prevalent types in lung cancer screenings (Supplementary Table S4). These systems maintained comparable detection performances between the standard and low-dose levels but showed sensitivity reductions of 0.07–0.12 at the ultra-low dose. CAD2 showed a lower but more consistent performance in detecting nodules under the reduction of doses. CAD2 exhibited lower yet dose-stable sensitivity across all protocols. For larger nodules (≥10 mm) and higher Lung-RADS categories, the DL-CAD systems showed improved sensitivity, with lower-dose-related variation. For the manual readings, they significantly outperformed the original CT and CAD2 for all subgroups (*p* < 0.05), particularly the detection of nodules at low- and ultra-low-dose CT scans (*p* < 0.05). In the multivariate logistic analysis of the independent predictors of nodule detection (Supplementary Table S8), nodule density, size, location, Lung-RADS, dose, kernel, and algorithm all influenced the sensitivity of the four DL-CAD systems and the manual readings (*p* < 0.05), and the effect of location for CT and CAD4 did not reach significance.

### 3.3. Volume Measurement

Table 5 presents the absolute volume errors (AVEs) of the four DL-CAD systems and the manual readings. Specifically, the automatic DL-CAD systems CAD1 and CAD4 demonstrated significantly higher average AVEs across all dose and reconstruction groups compared with the manual volumetry measurement (*p* < 0.05, Supplementary Table S6). Conversely, CAD3 and the semi-automatic CAD2 exhibited significantly lower overall AVEs than the manual volumetry measurement (*p* < 0.05, Supplementary Table S6). For the manual readings, the overall AVEs for the CT and VR were 14.98% ± 3.91% and 14.57% ± 4.76%, respectively, with no significant difference observed. In the subgroup analyses of the dose, kernel, and algorithm, the AVEs for the CT exhibited ranges from 12.63% to 18.82%, 13.95% to 16.02%, and 12.49% to 17.05%, respectively. For the VR, the AVEs ranged from 9.68% to 18.85%, 13.77% to 15.37%, and 11.79% to 16.29% across the same subgroups. A comparison of the AVEs between the manual reading methods and the DL-CAD systems reveals significant differences in performance.

In the volumetry measurement capabilities for the nodule size and density subgroups (Supplementary Table S7), the manual reading groups achieved AVEs < 15% for the standard or low doses, large nodules (>10 mm), solid or part-solid nodules, and Lung-RADS classifications 3 and 4B. In contrast, the four DL-CAD systems were less affected by the radiation dose in the volumetry measurement. Notably, CAD1 and CAD4 performed poorly in measuring nodules smaller than 5 mm (28.91% and 29.05%) or within a size range of 15–20 mm (56.16% and 45.36%), as well as those that were solid (27.24% and 24.65%) or part-solid (55.06% and 43.91%) in density and had Lung-RADS classifications of 3 (44.64% and 34.06%) and 4B (47.89% and 41.02%). These performances were opposite of what was observed for the manual measurements.

Further analysis using multivariate linear regression to determine the influencing factors for the AVE revealed that the AVEs were negatively associated with the GGNs (β = −0.21 and −0.22, *p* < 0.001 and *p* = 0.004) and Lung-RADS classifications of 3 (β = −0.31 and −0.30, *p* < 0.001), 4A (β = −0.33 and −0.32, *p* < 0.001), and 4B (β = −0.31 and −0.29, *p* < 0.001) for both CT and VR in the manual readings. For the DL-CAD systems, there were negative associations with GGNs and Lung-RADS 3, 4A, and 4B, as well as positive associations with PSN and a diameter of 10–15 mm. A low or ultra-low dose had negative effects on CAD1 and CAD3, a positive effect on CAD4, and no significant effect on CAD2. The sharp kernel of Br64 had negative effects on CAD1 and CAD2, while CAD3 and CAD4 were positively affected. Additionally, the reconstruction algorithm ADMIRE−5 was negatively associated with CAD3 and CAD4 (Supplementary Table S9).

### 3.4. Lung-RADS Classification

Table 6 shows the accuracies for Lung-RADS classification between the four DL-CAD systems and the manual readings. Among the DL-CAD systems, the lowest matching rates were found for categories 4A (47.38%) and 4B (46.71%, 33.84%, 42.12%) with CAD1 to CAD4, respectively. We further evaluated the performances in nodule-type classification for the three fully automatic DL-CAD systems (CAD1, CAD3, and CAD4; Supplementary Table S10). These systems correctly identified 28.31% (CAD1), 12.60% (CAD3), and 18.32% (CAD4) of all nodules; 35.15% (CAD1), 10.29% (CAD3), and 0.16% (CAD4) of the SNs; 42.35% (CAD1), 27.20% (CAD3), and 27.60% (CAD4) of the GGNs; and 0.89% (CAD1), 2.92% (CAD3), and 41.82% (CAD4) of the PSNs. These low nodule-type classification rates may account for the low Lung-RADS categories 4A and 4B classification rates observed for the DL-CAD systems. For the manual readings, the overall matching rates in the Lung-RADS classification varied from 80.39% to 90.83% for the original CT and from 78.77% to 88.20% for VR across the dose levels, which are higher than those observed for all of the DL-CAD systems.

Additionally, the study evaluated the influencing factors in Lung-RADS classification using multivariate logistical regression (Supplementary Table S11). For the manual readings, nodule density, diameter, Lung-RADS, dose level, kernel, and algorithm were all found to be influencing factors (*p* < 0.001). Similarly, for all four DL-CAD systems, nodule density, diameter, Lung-RADS, dose level, kernel, and algorithm were identified as influencing factors.

## 4. Discussion

Being able to accurately and efficiently detect and diagnose pulmonary nodules on CT scans is crucial for lung cancer screening and management. To facilitate the clinical applicability of the DL-CAD systems and update the benefits of post-processing techniques for manual reading in lung cancer screening, it is important to know the amount of variability associated with nodule characteristics, as well as imaging settings, including dose levels, and reconstruction kernels and algorithms. Our study noted that the four commercial DL-CAD systems exhibited consistent and high sensitivity in nodule detection, along with considerable volumetry measurement outcomes. This enhancement supports the potential use of DL-CAD in lung cancer screening and management. However, the primary shortcomings of the DL-CAD systems were observed in Lung-RADS classification, particularly in nodule-type classification. Compared with the DL-CAD systems, manual reading using VR outperformed the use of original CT images. VR achieved high sensitivity in nodule detection similar to the DL-CAD systems across radiation doses and reconstruction settings and demonstrated enhanced performances in specificity, accuracy, and F1 score. It also showed satisfactory performances in Lung-RADS classification and volume measurement and was less affected by poor image quality due to dose and reconstruction settings compared with the original CT images. This suggests that VR is a method with both high diagnostic performance and efficiency for lung cancer screening.

Although DL-CAD systems have been in use for several years, their performance in clinical settings is still considered suboptimal. Trust in these systems is challenged by concerns over relatively low specificity, variability in performance among different DL-CAD systems, and the largely unknown performance of DL-CAD under low-dose CT (LDCT) or ultra-low-dose CT (ULDCT) in lung cancer screening [11,12]. Our systematic summary of recent studies (2020–2025) on commercial DL-CAD systems for pulmonary nodule detection (Supplementary Table S12) suggests the alignment of our study with the predominant consensus that DL-CAD consistently demonstrates superior sensitivity in nodule detection, achieving an overall sensitivity of 92–95% in the current study, compared with the reported average sensitivity of 88%. Crucially, however, the existing literature predominantly focuses on the validation of a single DL-CAD system or fails to rigorously evaluate performance across varying radiation doses. Our study, however, demonstrates that the overall sensitivity of the majority of DL-CAD systems at low and ultra-low doses is notably high, reaching 95–97% and 85–91%, respectively. These figures are significantly better than those of traditional machine-learning-based CAD (ML-CAD) system and older versions of DL-CAD systems at the low- and ultra-low-dose levels [22,30]. Moreover, we reveal significant heterogeneity in both detection performance and robustness among commercial systems, a complexity that is substantially greater than previously documented and likely attributable to fundamental differences in algorithmic architectures and training data composition [17,31]. Also, it is important to note that each DL-CAD system exhibited optimal detection performance under different combinations of dose levels and reconstruction settings, with no specific pattern ensuring the best detection performance. Therefore, we recommend using the same DL-CAD tool for both lung cancer screening and follow-up sessions to maintain consistency. Generally, the evidence from the current study further supports the significant value of DL-CAD as a lung cancer screening tool across a broader range of clinical scenarios. The high sensitivity of the DL-CAD systems, even at low and ultra-low doses, suggests that they can play a crucial role in improving the efficiency and effectiveness of lung cancer screening programs.

For nodule management, precise volumetry measurement is essential for timely and correct diagnosis and treatment, particularly as the population engaged in major lung cancer screening programs continues to grow worldwide [32]. Our study reveals that CAD3 exhibited the lowest volumetry measurement error (9.66%), with AVEs below 10% under low- and ultra-low-dose scanning, Br64 kernel, and ADMIRE algorithm. The semi-automatic CAD2 had an overall AVE of 13.85%, which is similar to manual measurement using original CT (14.98%) and VR (14.57%). Given that the AVE results from these two systems were not subject to manual correction, this suggests an improving performance trend when compared with the results of older studies. However, CAD1 and CAD4 displayed lower volumetry measurement accuracy, with AVEs of 33.14% and 29.43%, respectively. Subgroup analysis of these two systems indicated that the primary source of the volumetry error was exclusively from the PSNs, resulting in AVEs of 55.06% and 43.91%, respectively. In contrast, the AVEs for the SNs and GGNs were relatively acceptable, with the majority of AVEs below 25%, which is the recommended threshold for distinguishing real growth from measurement error [33]. Additionally, for all four DL-CAD systems, high AVEs were observed for smaller nodules (≤5 mm), and, interestingly, the AVE decreased with reduced dose levels. Our study also identified multiple factors influencing the accuracy of the volumetry measurement, including nodule density, nodule size, Lung-RADS classification, dose, and kernel. The algorithm was identified as an influencing factor in only half of the DL-CAD systems, which is partly consistent with previous findings [13].

Currently, DL-CAD systems are primarily used as the second or concurrent reader in Lung-RADS classification tasks. Several studies have shown that the use of semi-automatic measurement from CAD can improve interobserver agreement in Lung-RADS classification. However, the majority of these studies report that the added value of CAD is minimal [32,34]. In our study, standalone DL-CAD showed relatively low accuracy in performing Lung-RADS classification tasks, with the accuracy ranging from 55% to 69% for three automatic DL-CAD systems and 81% for the semi-automatic DL-CAD system, which partly accords with previous findings [35]. The primary error in Lung-RADS classification for various DL-CAD systems occurred in the nodule-density-type classification, where each automatic DL-CAD system reported lower accuracy. More specifically, we observed systematic errors in classifying solid nodules as calcification, potentially leading to a down-shift classification according to Lung-RADS v2022. Additionally, there were misclassifications of GGNs as PSNs for all automatic DL-CAD systems due to image noise, which could potentially lead to an up-shift in Lung-RADS classification. We also noted that CAD3 tended to categorize most high-grade PSNs as 4X, and CAD4 classified most PSNs above a Lung-RADS category 3 as GGNs. Conversely, although semi-automatic CAD2 did not provide the function of nodule-density-type classification, we did not observe systematic errors in classifying GGNs and PSNs, as reported by Shu et al. [36]. Therefore, the mismatches in Lung-RADS classification with CAD2 were solely from the bias in semi-automatic measurement, which may potentially explain the similar results for CAD2 with the two manual methods. It is important to note that the included artificial nodules were smooth and spherical, which might include potential overestimation of the performance of DL-CAD systems for standalone classification of Lung-RADS. Nonetheless, these findings support the current recommendation that CAD should only be used as a second or concurrent reader for nodule diagnosis.

Despite the trust issues of radiologists regarding DL-CAD systems being expected to be relieved with the substantial advancement of AI in medicine, there are also challenges related to human–AI interaction [37,38]. Often, DL-CAD systems are not integrated into the PACS workstation, requiring radiologists to switch among software and repeat the image reading process manually, as DL-CAD systems do not always present nodules in the order preferred by individual radiologists. This has made manual reading still more preferable for the majority of radiologists. Post-processing techniques, which are normally pre-integrated into a PACS workstation, are more accessible for manual interpretation. Studies have shown that the use of post-processing techniques can significantly improve the detection rates for pulmonary nodules [28,29]. However, these techniques display only the maximal or minimal intensity voxels, limiting their ability to show nodules with various densities and provide relevant information in complex anatomic contexts necessary for size or volumetry measurements of lesions. Conversely, VR displays every voxel with an assigned opacity and pseudo color, significantly helping to reduce perception errors caused by so-called anatomical noise from normal lung structures, such as vascular structures, airways, and the interstitium [20]. Prior studies have already suggested the diagnostic potential of VR for pulmonary nodules, but there is a relative lack of follow-up evidence. In our study, VR showed superior diagnostic performance over original CT [19,20]. Firstly, compared with original CT, VR demonstrated significant improvements in diagnostic performance measures, including sensitivity (0.92 vs. 0.72, *p* < 0.001), specificity (0.98 vs. 0.81, *p* < 0.001), accuracy (0.95 vs. 0.77, *p* < 0.001), and F1 score (0.94 vs. 0.79, *p* < 0.001). These improvements were observed across radiation-dose levels, and reconstruction kernels and algorithms. Secondly, VR showed better sensitivity over original CT for nodules with a diameter under 15 mm, solid or pure ground-glass density, and Lung-RADS classifications of 2 and 4B, with differences reaching significance under low-dose and ultra-low-dose scanning. Lastly, the added value of VR is especially considerable in less-experienced radiologists, and the image quality and reading speed for VR are superior to those for original CT images.

The evaluated performances of the four DL-CAD systems and VR directly address two critical clinical objectives fundamental to lung cancer screening, specifically maximizing the early detection of potentially malignant pulmonary nodules and enabling risk stratification primarily via precise volume measurement and Lung-RADS classification for personalized management guidance [39,40]. Our findings demonstrate that both DL-CAD systems and VR maintain high detection sensitivity under low- and ultra-low-dose conditions, particularly for diagnostically challenging sub-centimeter nodules prevalent in lung screening, significantly outperforming conventional CT reading at reduced scanning levels. This capability facilitates timely identification of early-stage malignancies, reducing the risks associated with diagnostic delays. However, substantial limitations remain in current DL-CAD systems for independently performing accurate Lung-RADS classification, especially for part-solid nodules, for which precise characterization of internal components remains challenging. Such inaccuracies may compromise risk stratification, potentially misclassifying high-risk category 4A/4B nodules as lower risk or, conversely, adversely influencing subsequent clinical decisions regarding interventions [41]. Consequently, while valuable for detection, DL-CAD currently functions best as an adjunctive tool such as a second reader, with final classification requiring radiologist integration of CAD outputs and primary image interpretation. These results underscore the need for algorithmic improvements in complex nodule characterization to enhance clinical decision support. Furthermore, specific DL-CAD implementations and VR-assisted volumetry achieved stable measurements with AVEs below 15% across radiation doses. This reliability in serial LDCT/ULDCT surveillance is essential for monitoring nodule growth, which is the key malignancy indicator, particularly for small nodules, thereby distinguishing benign lesions from those warranting prompt intervention [42]. Finally, VR optimized the clinical workflow by reducing the interpretation time and improving the perceived image quality, especially for less-experienced readers under suboptimal imaging conditions. Its efficiency positions VR as a practical frontline screening tool for rapid triage of high-risk nodules requiring detailed assessment.

Our study has several limitations. Firstly, this study is based on a phantom model, which inevitably introduces differences between the results and those from real clinical patients. Nonetheless, the promising results derived from this study have prompted our institution to advance preparations for clinical trials aimed at further validating the diagnostic efficacy of VR and DL-CAD systems. Secondly, while our study encompassed a broader spectrum of simulated pulmonary nodules, the uniform spherical nature of these nodules may, to a certain extent, overstate the efficacy of manual reading methodologies. Consequently, it is imperative to replicate this study within a clinical framework to ascertain the generalizability of our findings. Lastly, it is important to note that the conclusions drawn from this study are pertinent to specific DL-CAD software and versions, as variations in software or versioning could lead to divergent diagnostic outcomes for pulmonary nodules.

Our study demonstrated that DL-CAD systems as standalone readers are capable of accurately detecting pulmonary nodules and exhibit acceptable performance in volumetry measurement under different scanning dose levels and reconstruction settings of the kernel and algorithms. This emphasizes the potential utility of DL-CAD systems in aiding radiologists during LDCT or ULDCT lung cancer screenings. However, it is also evident that there are systemic deficiencies in Lung-RADS classification and nodule-type identification within current DL-CAD systems that warrant further attention and improvement. For manual reading, VR is shown to enhance diagnostic precision and efficiency compared with original CT, particularly for less-experienced radiologists and in scenarios where the scanning dose or image quality is compromised. This suggests that VR could emerge as a valuable diagnostic adjunct in lung cancer screening protocols.

## Figures and Tables

**Table 1:** Overview of the imaging protocols.

	Standard Dose (*n* = 360)	Low Dose (*n* = 360)	Ultra-Low Dose (*n* = 360)
#Datasets	360	360	360
Scanner	SOMATOMA Force		
kVp	120 kVp	100 kVp	Sn100 kVp
mAs	100 mAs	50 mAs	45 mAs
CTDIvol (mGy)	5.71 ± 0.21	1.76 ± 0.08	0.15 ± 0.01
mSv	2.82 ± 0.11	0.87 ± 0.04	0.07 ± 0.01
Reconstruction kernel	Br40, Br64		
Reconstruction algorithm	Filtered back projection, iterative reconstruction (ADMIRE−3, ADMIRE−5)		
Slice thickness	1 mm		

mSv = millisievert.

**Table 2:** Overview of the involved DL-CAD systems.

	CAD1	CAD2	CAD3	CAD4
Product	InferRead CT Lung	Lung CAD	uAI-ChestCare	LungDoc
Vendor	InferVision Medical Health	Siemens Healthcare	United Imaging Healthcare	Shukun Technology
Country	China	German	China	China
Version	Ifocr6.1.5.4	VD20A	R001.0.1.42690	V8.7.616.1
Model	DenseNet + modified Faster R-CNN	3D CNN + cascaded CNN	cascade FPN + VB-Net	modified FPN + UNet + ResNet
License	NMPA (II), MDR CE, FDA, PMDA	FDA, MDR CE, PMDA	MDR CE, NMPA (III)	MDR CE, NMPA (III)

**Table 3:** Nodule detection.

Metric	Model	Dose			Kernel		Algorithm			Total
		SDCT	LDCT	ULDCT	Br40	Br64	FBP	ADMIRE−3	ADMIRE−5	
Sensitivity	CAD1	0.97	0.96	0.88	0.95	0.91	0.91	0.94	0.95	0.93
	CAD2	0.63 *	0.67 *	0.73	0.73 *	0.63 *	0.69 *	0.67 *	0.67 *	0.68 *
	CAD3	0.96	0.95	0.85	0.94	0.90	0.89	0.93	0.94	0.92
	CAD4	0.98	0.97	0.91	0.97	0.93	0.93	0.96	0.97	0.95
	CT	0.82 *	0.73 *	0.62 *	0.77 *	0.68 *	0.63 *	0.73 *	0.81	0.72 *
	VR	0.96	0.92	0.88	0.94	0.90	0.89	0.92	0.95	0.92
Specificity	CAD1	0.89	0.80 *	0.66 *	0.90	0.66 *	0.72 *	0.79 *	0.84 *	0.78 *
	CAD2	0.92	0.95	0.96	0.94	0.95	0.94	0.95	0.94	0.94
	CAD3	0.92	0.94	0.84 *	0.99	0.82 *	0.86 *	0.90	0.94	0.90
	CAD4	0.80 *	0.84 *	0.91	0.86	0.84 *	0.82 *	0.87 *	0.86	0.85 *
	CT	0.89	0.82 *	0.73 *	0.84 *	0.79 *	0.78 *	0.83 *	0.83 *	0.81 *
	VR	0.98	0.97	0.97	0.98	0.97	0.97	0.98	0.98	0.98
Accuracy	CAD1	0.92	0.86	0.74 *	0.92	0.75 *	0.79	0.84 *	0.88	0.84 *
	CAD2	0.82 *	0.85 *	0.88	0.86 *	0.83 *	0.85	0.85 *	0.84 *	0.85 *
	CAD3	0.93	0.94	0.84	0.97	0.85	0.87	0.91	0.94	0.91
	CAD4	0.87 *	0.89	0.91	0.90	0.87	0.86	0.90	0.90	0.89
	CT	0.85 *	0.77 *	0.68 *	0.81 *	0.73 *	0.70 *	0.77 *	0.83 *	0.77 *
	VR	0.97	0.95	0.93	0.96	0.94	0.94	0.95	0.97	0.95

Group comparisons were performed with VR as the reference using the chi-square test. A 2-sided *p* < 0.05 was considered statistically significant following Bonferroni correction for multiple comparisons. * A significant difference was observed. SDCT = standard-dose computed tomography; LDCT = low-dose computed tomography; ULDCT = ultra-low-dose computed tomography; VR = volume rendering. FBP = filtered back projection.

**Table 4:** Subgroup analysis of the nodule detection.

Model	Dose	Size				Density			Lung-RADS			
		≤5 mm	5–10 mm	10–15 mm	15–20 mm	SN	GGN	PSN	2	3	4A	4B
CAD1		0.82 *	0.92	0.96	0.99	0.97	0.82	0.99	0.88	0.99	0.98	0.99
	Standard	0.87	0.97	1.00	1.00	0.98	0.93	1.00	0.95	1.00	0.98	1.00
	Low	0.83 #	0.96	0.98	1.00	0.98	0.87	1.00	0.92 #	1.00	0.99	1.00
	Ultra-low	0.76 #	0.82	0.90	0.97	0.95	0.65 #	0.97	0.77	0.97	0.99	0.97
CAD2		0.50 *	0.70 *	0.77 *	0.70 *	0.81 *	0.50 *	0.71 *	0.53 *	0.75 *	0.88 *	0.78 *
	Standard	0.48 #	0.68 #	0.71 #	0.60 #	0.79 #	0.46 #	0.60 #	0.50 #	0.65 #	0.84 #	0.71 #
	Low	0.47 #	0.71 #	0.76 #	0.71 #	0.80 #	0.51 #	0.69	0.53 #	0.72 #	0.89 #	0.77 #
	Ultra-low	0.55	0.69 #	0.85 #	0.81 #	0.85	0.52 #	0.82 #	0.56 #	0.87 #	0.92	0.85 #
CAD3		0.84 *	0.92	0.97	0.99	0.96	0.86	0.99	0.85	0.99	0.99	1.00
	Standard	0.89	0.97	0.99	1.00	0.95	0.96	1.00	0.93	1.00	1.00	1.00
	Low	0.86	0.95	0.99	1.00	0.96	0.92	1.00	0.89	1.00	1.00	1.00
	Ultra-low	0.77 #	0.85	0.92	0.98	0.95	0.71	0.98	0.73	0.98	0.97	1.00
CAD4		0.90 *	0.93	0.97	0.99	0.98	0.88	0.99	0.85	0.99	0.99	0.99
	Standard	0.93	0.99	0.99	0.99	0.99	0.97	0.99	0.93	0.99	1.00	1.00
	Low	0.91 #	0.96	0.98	1.00	1.00	0.91	1.00	0.89	1.00	1.00	1.00
	Ultra-low	0.86 #	0.86	0.94	0.98	0.97	0.78	0.98	0.73	0.99	0.97	0.97
CT		0.26 *	0.73 *	0.85 *	0.93	0.68 *	0.72 *	0.91	0.54 *	0.91	0.90	0.83 *
	Standard	0.34 #	0.87	0.93	0.99	0.76 #	0.82 #	0.98	0.66 #	0.98	0.96	0.94
	Low	0.26 #	0.71 #	0.86 #	0.97	0.67 #	0.73 #	0.97	0.53 #	0.98	0.90 #	0.80 #
	Ultra-low	0.17 #	0.62 #	0.76 #	0.83 #	0.61 #	0.62 #	0.79 #	0.42 #	0.77 #	0.83 #	0.74 #
VR		0.71	0.94	0.98	1.00	0.93	0.86	1.00	0.84	1.00	1.00	0.99
	Standard	0.84	0.97	1.00	1.00	0.96	0.94	1.00	0.93	1.00	0.99	1.00
	Low	0.70	0.94	0.98	1.00	0.93	0.86	1.00	0.84	1.00	1.00	0.99
	Ultra-low	0.59	0.90	0.97	0.99	0.89	0.78	0.99	0.76	1.00	0.99	0.98

Group comparisons are performed with VR as the reference using a chi-square test. A 2-sided *p* < 0.05 was considered statistically significant following Bonferroni correction for multiple comparisons. * A significant difference was observed. # A significant difference was observed at the same scanning dose. Lung-RADS = Lung CT Screening Reporting and Data Systems; VR = volume rendering; CT = computed tomography; SNs = solid nodules; GGNs = ground-glass nodules; PSNs = part-solid nodules.

**Table 5:** Absolute volume error.

Model	Dose (%)			Kernel (%)		Algorithm (%)			Total (%)
	SDCT	LDCT	ULDCT	Br40	Br64	FBP	ADMIRE−3	ADMIRE−5	
CAD1	**37.32 ± 3.53**	**32.63 ± 4.95**	**29.45 ± 8.94**	**33.37 ± 6.74**	**32.91 ± 7.15**	**32.83 ± 8.56**	**32.12 ± 5.83**	**34.46 ± 6.62**	**33.14 ± 6.74**
CAD2	15.74 ± 1.95	13.39 ± 1.33	12.43 ± 2.43	15.35 ± 1.81	12.36 ± 1.79	13.74 ± 2.52	13.39 ± 2.51	14.42 ± 2.26	**13.85 ± 2.33**
CAD3	13.34 ± 15.73	**6.90 ± 3.82**	8.73 ± 5.89	**8.30 ± 13.63**	11.02 ± 3.45	15.27 ± 15.50	**6.95 ± 3.97**	**6.76 ± 3.19**	**9.66 ± 9.75**
CAD4	**26.47 ± 9.56**	**32.63 ± 4.95**	**29.18 ± 9.16**	**26.41 ± 8.18**	**32.45 ± 7.19**	**29.59 ± 9.81**	**28.04 ± 6.80**	**30.66 ± 8.71**	**29.43 ± 8.09**
CT	12.63 ± 3.78	**13.50 ± 1.58**	18.82 ± 2.90	**16.02 ± 3.41**	13.95 ± 4.31	17.05 ± 3.77	15.40 ± 3.83	12.49 ± 3.22	14.98 ± 3.91
VR	9.68 ± 3.38	15.18 ± 3.13	18.85 ± 4.58	15.37 ± 4.11	13.77 ± 6.33	16.29 ± 6.02	15.64 ± 5.47	11.79 ± 3.59	14.57 ± 5.24

Data are the percentages presented as the mean ± SDs. Group comparisons were performed with VR as the reference, with repeated measures analysis of the variance with Greenhouse–Geisser correction followed by a post hoc test. A 2-sided *p* < 0.05 was considered statistically significant following Bonferroni correction for multiple comparisons. Bold text indicates statistical significance. VR = volume rendering; SDCT = standard-dose computed tomography; LDCT = low-dose computed tomography; ULDCT = ultra-low-dose computed tomography; FBP = filtered back projection; CT = computed tomography; VR = volume rendering.

**Table 6:** Lung-RADS classification accuracy.

Model	Dose	2 (%)	3 (%)	4A (%)	4B (%)	Total (%)
CAD1		50.53 *	74.91	47.38 *	73.40	55.23 *
	Standard	49.82 #	77.33 #	47.09 #	78.79	55.36 #
	Low	52.41 #	62.00 #	42.00 #	62.12 #	51.53 #
	Ultra-low	49.36 #	85.39	53.05 #	79.29	58.78 #
CAD2		73.14	84.52	94.12	46.71 *	80.75
	Standard	73.91 #	94.38	93.04	35.62 #	80.75
	Low	74.58	91.56	94.20 #	59.95 #	83.71
	Ultra-low	70.92	67.62 #	95.12 #	44.57 #	77.17
CAD3		65.76 *	41.56 *	57.53 *	33.84 *	57.00 *
	Standard	70.42 #	40.67 #	63.27 #	33.33 #	60.97 #
	Low	68.91 #	41.33 #	54.23 #	40.91 #	58.44 #
	Ultra-low	57.96 #	42.67 #	55.10 #	27.27 #	51.60 #
CAD4		85.48	45.12 *	65.71 *	42.12 *	69.42 *
	Standard	81.36	54.67 #	65.99 #	50.00 #	70.29 #
	Low	81.40	47.15 #	65.65 #	46.97 #	69.15 #
	Ultra-low	93.68 #	33.54 #	65.51 #	29.39 #	68.81
CT		86.02	83.26	85.41	88.33	85.51
	Standard	95.45	87.50	88.64	87.50 #	90.83
	Low	85.45	83.33	85.11	90.00	85.29
	Ultra-low	77.14	78.95	82.50	87.50 #	80.39
VR		84.53	86.44	81.25	77.88	83.47
	Standard	92.21	92.00	83.33	72.73	88.20
	Low	84.81	84.00	79.17	90.91	83.44
	Ultra-low	76.56	83.33	81.25	70.00	78.77

Group comparisons were performed with VR as the reference using chi-square tests. A 2-sided *p* < 0.05 was considered statistically significant following Bonferroni correction for multiple comparisons. * A significant difference was observed. # A significant difference was observed at the same scanning dose. CT = computed tomography; VR = volume rendering.

## Data Availability

The datasets generated or analyzed during the study are available from the corresponding author upon reasonable request.

## Supplementary Materials

The following supporting information can be downloaded at: https://www.mdpi.com/article/10.3390/diagnostics15131623/s1, Table S1. Characteristics of the simulated pulmonary nodules; Table S2. Comparison of diagnostic performance of four DL-CAD systems and manual reading; Table S3. Comparison of diagnostic performance for manual reading; Table S4. Comparison of sensitivity of four DL-CAD systems and manual reading in nodule subgroups; Table S5. Reading Time; Table S6. Comparison of Absolute Volume Error of four DL-CAD systems and manual reading; Table S7. Subgroup Analysis of Absolute Volume Error; Table S8. Logistic Regression Analysis of Influencing Factors Associated with Detection Rates; Table S9. Multivariable Generalized Linear Mixed Model for Influencing Factors for AVE; Table S10. Performance of nodule density type classification of three automatic DL-CAD; Table S11. Logistic Regression Analysis of Influencing Factors Associated with Lung-RADS Classification; Table S12. Literature summary of commercial DL-CAD systems for pulmonary nodule detection during the past five years (2021–2025); Figure S1. Comparison of pulmonary nodule visualization using axial CT, maximum intensity projection (MIP, slab thickness of 10 mm), and volume rendering (VR, slab thickness of 10 mm) techniques under low dose computed tomography (LDCT), and ultra-low dose computed tomography (ULDCT) protocols; Figure S2. Opacity-CT value curve setting of VR; Figure S3 shows subjective image quality ratings of two readers for the different scanning protocols. Subjective image quality was graded on a 5-point Likert scale: 1 = nondiagnostic image quality, strong artifacts, insufficient for diagnostic purposes score; 2 = severe artifacts with uncertainty about the evaluation; 3 = moderate artefacts with restricted assessment; 4 = slight artifacts with unrestricted diagnostic image evaluation possible; and 5 = excellent image quality, no artifacts.

## References

[1] Leiter A., Veluswamy R.R., Wisnivesky J.P. (2023). The Global Burden of Lung Cancer: Current Status and Future Trends. Nat. Rev. Clin. Oncol..

[2] Henschke C.I., Yip R., Shaham D., Markowitz S., Cervera Deval J., Zulueta J.J., Seijo L.M., Aylesworth C., Klingler K., Andaz S. (2023). A 20-Year Follow-up of the International Early Lung Cancer Action Program (I-ELCAP). Radiology.

[3] de Koning H.J., van der Aalst C.M., de Jong P.A., Scholten E.T., Nackaerts K., Heuvelmans M.A., Lammers J.-W.J., Weenink C., Yousaf-Khan U., Horeweg N. (2020). Reduced Lung-Cancer Mortality with Volume CT Screening in a Randomized Trial. N. Engl. J. Med..

[4] Wood D.E., Kazerooni E.A., Baum S.L., Eapen G.A., Ettinger D.S., Hou L., Jackman D.M., Klippenstein D., Kumar R., Lackner R.P. (2018). Lung Cancer Screening, Version 3.2018, NCCN Clinical Practice Guidelines in Oncology. J. Natl. Compr. Cancer Netw..

[5] McDonald R.J., Schwartz K.M., Eckel L.J., Diehn F.E., Hunt C.H., Bartholmai B.J., Erickson B.J., Kallmes D.F. (2015). The Effects of Changes in Utilization and Technological Advancements of Cross-Sectional Imaging on Radiologist Workload. Acad. Radiol..

[6] Smith-Bindman R., Miglioretti D.L., Johnson E., Lee C., Feigelson H.S., Flynn M., Greenlee R.T., Kruger R.L., Hornbrook M.C., Roblin D. (2012). Use of Diagnostic Imaging Studies and Associated Radiation Exposure for Patients Enrolled in Large Integrated Health Care Systems 1996–2010. JAMA.

[7] Smith-Bindman R., Miglioretti D.L., Larson E.B. (2008). Rising Use of Diagnostic Medical Imaging in a Large Integrated Health System. Health Aff..

[8] Hosny A., Parmar C., Quackenbush J., Schwartz L.H., Aerts H.J.W.L. (2018). Artificial Intelligence in Radiology. Nat. Rev. Cancer.

[9] van Leeuwen K.G., Schalekamp S., Rutten M.J.C.M., van Ginneken B., de Rooij M. (2021). Artificial Intelligence in Radiology: 100 Commercially Available Products and Their Scientific Evidence. Eur. Radiol..

[10] Cressman S., Peacock S.J., Tammemägi M.C., Evans W.K., Leighl N.B., Goffin J.R., Tremblay A., Liu G., Manos D., MacEachern P. (2017). The Cost-Effectiveness of High-Risk Lung Cancer Screening and Drivers of Program Efficiency. J. Thorac. Oncol..

[11] Gu Y., Chi J., Liu J., Yang L., Zhang B., Yu D., Zhao Y., Lu X. (2021). A Survey of Computer-Aided Diagnosis of Lung Nodules from CT Scans Using Deep Learning. Comput. Biol. Med..

[12] Schreuder A., Scholten E.T., van Ginneken B., Jacobs C. (2021). Artificial Intelligence for Detection and Characterization of Pulmonary Nodules in Lung Cancer CT Screening: Ready for Practice?. Transl. Lung Cancer R.

[13] Guedes Pinto E., Penha D., Ravara S., Monaghan C., Hochhegger B., Marchiori E., Taborda-Barata L., Irion K. (2023). Factors Influencing the Outcome of Volumetry Tools for Pulmonary Nodule Analysis: A Systematic Review and Attempted Meta-Analysis. Insights Imaging.

[14] Park S., Lee S.M., Kim W., Park H., Jung K.-H., Do K.-H., Seo J.B. (2021). Computer-Aided Detection of Subsolid Nodules at Chest CT: Improved Performance with Deep Learning-Based CT Section Thickness Reduction. Radiology.

[15] Schwyzer M., Messerli M., Eberhard M., Skawran S., Martini K., Frauenfelder T. (2022). Impact of Dose Reduction and Iterative Reconstruction Algorithm on the Detectability of Pulmonary Nodules by Artificial Intelligence. Diagn. Interv. Imaging.

[16] Peters A.A., Christe A., von Stackelberg O., Pohl M., Kauczor H.-U., Heußel C.P., Wielpütz M.O., Ebner L. (2023). “Will I Change Nodule Management Recommendations If I Change My CAD System?”—Impact of Volumetric Deviation between Different CAD Systems on Lesion Management. Eur. Radiol..

[17] Mohammad B.A., Brennan P.C., Mello-Thoms C. (2017). A Review of Lung Cancer Screening and the Role of Computer-Aided Detection. Clin. Radiol..

[18] Ji Y., Zhang T., Yang L., Wang X., Qi L., Tan F., Daemen J.H.T., de Loos E.R., Qiu B., Gao S. (2021). The Effectiveness of Three-Dimensional Reconstruction in the Localization of Multiple Nodules in Lung Specimens: A Prospective Cohort Study. Transl. Lung Cancer Res..

[19] Li W.-J., Chu Z.-G., Li D., Jing W.-W., Shi Q.-L., Lv F.-J. (2024). Accuracy of Solid Portion Size Measured on Multiplanar Volume Rendering Images for Assessing Invasiveness in Lung Adenocarcinoma Manifesting as Subsolid Nodules. Quant. Imaging Med. Surg..

[20] Peloschek P., Sailer J., Weber M., Herold C.J., Prokop M., Schaefer-Prokop C. (2007). Pulmonary Nodules: Sensitivity of Maximum Intensity Projection versus That of Volume Rendering of 3D Multidetector CT Data. Radiology.

[21] Hop J.F., Walstra A.N.H., Pelgrim G.-J., Xie X., Panneman N.A., Schurink N.W., Faby S., van Straten M., de Bock G.H., Vliegenthart R. (2023). Detectability and Volumetric Accuracy of Pulmonary Nodules in Low-Dose Photon-Counting Detector Computed Tomography: An Anthropomorphic Phantom Study. Diagnostics.

[22] Fu B., Wang G., Wu M., Li W., Zheng Y., Chu Z., Lv F. (2020). Influence of CT Effective Dose and Convolution Kernel on the Detection of Pulmonary Nodules in Different Artificial Intelligence Software Systems: A Phantom Study. Eur. J. Radiol..

[23] Liu K., Li Q., Ma J., Zhou Z., Sun M., Deng Y., Tu W., Wang Y., Fan L., Xia C. (2019). Evaluating a Fully Automated Pulmonary Nodule Detection Approach and Its Impact on Radiologist Performance. Radiol. Artif. Intell..

[24] Perl R.M., Grimmer R., Hepp T., Horger M.S. (2021). Can a Novel Deep Neural Network Improve the Computer-Aided Detection of Solid Pulmonary Nodules and the Rate of False-Positive Findings in Comparison to an Established Machine Learning Computer-Aided Detection?. Investig. Radiol..

[25] Jiang Q., Sun H., Chen Q., Huang Y., Li Q., Tian J., Zheng C., Mao X., Jiang X., Cheng Y. (2025). High-Resolution Computed Tomography with 1024-Matrix for Artificial Intelligence-Based Computer-Aided Diagnosis in the Evaluation of Pulmonary Nodules. J. Thorac. Dis..

[26] Chen L., Gu D., Chen Y., Shao Y., Cao X., Liu G., Gao Y., Wang Q., Shen D. (2021). An Artificial-Intelligence Lung Imaging Analysis System (ALIAS) for Population-Based Nodule Computing in CT Scans. Comput. Med. Imaging Graph..

[27] Ebner L., Roos J.E., Christensen J.D., Dobrocky T., Leidolt L., Brela B., Obmann V.C., Joy S., Huber A., Christe A. (2016). Maximum-Intensity-Projection and Computer-Aided-Detection Algorithms as Stand-Alone Reader Devices in Lung Cancer Screening Using Different Dose Levels and Reconstruction Kernels. Am. J. Roentgenol..

[28] Fishman E.K., Ney D.R., Heath D.G., Corl F.M., Horton K.M., Johnson P.T. (2006). Volume Rendering versus Maximum Intensity Projection in CT Angiography: What Works Best, When, and Why. RadioGraphics.

[29] Johnson P.T., Fishman E.K. (2018). Enhancing Image Quality in the Era of Radiation Dose Reduction: Postprocessing Techniques for Body CT. J. Am. Coll. Radiol..

[30] Kozuka T., Matsukubo Y., Kadoba T., Oda T., Suzuki A., Hyodo T., Im S., Kaida H., Yagyu Y., Tsurusaki M. (2020). Efficiency of a Computer-Aided Diagnosis (CAD) System with Deep Learning in Detection of Pulmonary Nodules on 1-Mm-Thick Images of Computed Tomography. Jpn. J. Radiol..

[31] Blazis S.P., Dieckens D.B.M., Linsen P.V.M., Martins Jarnalo C.O. (2021). Effect of CT Reconstruction Settings on the Performance of a Deep Learning Based Lung Nodule CAD System. Eur. J. Radiol..

[32] Gierada D.S., Rydzak C.E., Zei M., Rhea L. (2020). Improved Interobserver Agreement on Lung-RADS Classification of Solid Nodules Using Semiautomated CT Volumetry. Radiology.

[33] Devaraj A., van Ginneken B., Nair A., Baldwin D. (2017). Use of Volumetry for Lung Nodule Management: Theory and Practice. Radiology.

[34] Park S., Park H., Lee S.M., Ahn Y., Kim W., Jung K., Seo J.B. (2022). Application of Computer-Aided Diagnosis for Lung-RADS Categorization in CT Screening for Lung Cancer: Effect on Inter-Reader Agreement. Eur. Radiol..

[35] Peters A.A., Wiescholek N., Müller M., Klaus J., Strodka F., Macek A., Primetis E., Drakopulos D., Huber A.T., Obmann V.C. (2024). Impact of Artificial Intelligence Assistance on Pulmonary Nodule Detection and Localization in Chest CT: A Comparative Study among Radiologists of Varying Experience Levels. Sci. Rep..

[36] Shu J., Wen D., Xu Z., Meng X., Zhang Z., Lin S., Zheng M. (2022). Improved Interobserver Agreement on Nodule Type and Lung-RADS Classification of Subsolid Nodules Using Computer-Aided Solid Component Measurement. Eur. J. Radiol..

[37] Gaube S., Suresh H., Raue M., Merritt A., Berkowitz S.J., Lermer E., Coughlin J.F., Guttag J.V., Colak E., Ghassemi M. (2021). Do as AI Say: Susceptibility in Deployment of Clinical Decision-Aids. Npj Digit. Med..

[38] Lee J.H., Hong H., Nam G., Hwang E.J., Park C.M. (2023). Effect of Human-AI Interaction on Detection of Malignant Lung Nodules on Chest Radiographs. Radiology.

[39] Bankier A.A., MacMahon H., Goo J.M., Rubin G.D., Schaefer-Prokop C.M., Naidich D.P. (2017). Recommendations for Measuring Pulmonary Nodules at CT: A Statement from the Fleischner Society. Radiology.

[40] Mazzone P.J., Lam L. (2022). Evaluating the Patient with a Pulmonary Nodule: A Review. JAMA.

[41] Christensen J., Prosper A.E., Wu C.C., Chung J., Lee E., Elicker B., Hunsaker A.R., Petranovic M., Sandler K.L., Stiles B. (2024). ACR Lung-RADS V2022: Assessment Categories and Management Recommendations. Chest.

[42] Adams S.J., Stone E., Baldwin D.R., Vliegenthart R., Lee P., Fintelmann F.J. (2023). Lung Cancer Screening. Lancet.

