# Supplementary material for: Performance Evaluation of Four Deep Learning-Based CAD Systems and Manual Reading for Pulmonary Nodules Detection, Volume Measurement, and Lung-RADS Classification Under Varying Radiation Doses and Reconstruction Methods
Source: Diagnostics (Basel). 2025 Jun 26;15(13):1623. doi: 10.3390/diagnostics15131623 (PMC12248771; doi:10.3390/diagnostics15131623)

Supplementary material

Performance metrics formula

$$\text{Sensitivity} = \text{True Positives} / (\text{True Positives} + \text{False Negatives})$$

$$\text{Specificity} = \text{True Negatives} / (\text{True Negatives} + \text{False Positives})$$

$$\text{Accuracy} = (\text{True Positives} + \text{True Negatives}) / (\text{True Positives} + \text{True Negatives} + \text{False Positives} + \text{False Negatives})$$

$$\text{Precision} = \text{True Positives} / (\text{True Positives} + \text{False Positives})$$

$$\text{F1 score} = 2 * (\text{Precision} * \text{Sensitivity}) / (\text{Precision} + \text{Sensitivity})$$

$$\text{AVE} = |\text{Measured volume} - \text{True volume}| / \text{True volume}$$

Table S1. Characteristics of the simulated pulmonary nodules

Density	Diameter	Volume	HU
SN	3mm	$14.14mm^3$	100
SN	5mm	$65.45mm^3$	100
SN	8mm	$268.08mm^3$	100
SN	10mm	$523.60mm^3$	100
SN	12mm	$904.78mm^3$	100
GGN	15mm	$1767.15mm^3$	-750
GGN	15mm	$1767.15mm^3$	-650
GGN	15mm	$1767.15mm^3$	-550
GGN	15mm	$1767.15mm^3$	-450
GGN	15mm	$1767.15mm^3$	-350
GGN	5mm	$65.45mm^3$	-800
GGN	8mm	$268.08mm^3$	-800
GGN	10mm	$523.60mm^3$	-800
GGN	12mm	$904.78mm^3$	-800
GGN	3mm	$14.14mm^3$	-630
GGN	5mm	$65.45mm^3$	-630
GGN	8mm	$268.08mm^3$	-630
GGN	10mm	$523.60mm^3$	-630
GGN	12mm	$904.78mm^3$	-630
PSN	15mm (5mm)	$1767.15mm^3$ ($65.45mm^3$)	-650(-50)
PSN	15mm (5mm)	$1767.15mm^3$ ($65.45mm^3$)	-650(0)
PSN	15mm (5mm)	$1767.15mm^3$ ($65.45mm^3$)	-650(50)
PSN	20mm (3mm)	$4188.79mm^3$ ($14.14mm^3$)	-650(0)
PSN	20mm (5mm)	$4188.79mm^3$ ($65.45mm^3$)	-650(0)
PSN	20mm (7mm)	$4188.79mm^3$ ($179.59mm^3$)	-650(0)
PSN	20mm (9mm)	$4188.79mm^3$ ($381.70mm^3$)	-650(0)
PSN	20mm (3mm+5mm)	$4188.79mm^3$ ($14.14mm^3+65.45mm^3$)	-650(0)
PSN	20mm (5mm+7mm)	$4188.79mm^3$ ($65.45mm^3+179.59mm^3$)	-650(0)

All simulated nodules are smooth,spherical nodules. Diameter, volume and HU information of solid component are presented in parentheses. SN, solid nodule; GGN, ground-glass nodule; PSN, part-solid nodule; HU, Hounsfield unit.

Table S2. Comparison of diagnostic performance of four DL-CAD systems and manual reading.

	Group comparison	Dose			Kernel		Algorithm			Total
		SDCT	LDCT	ULDCT	Br40	Br64	FBP	ADMIRE-3	ADMIRE-5	
	CAD1 vs CT	< .001	< .001	< .001	< .001	< .001	< .001	< .001	< .001	< .001
	CAD2 vs CT	< .001	0.048	< .001	0.563	0.268	0.065	0.048	< .001	0.740
	CAD3 vs CT	< .001	< .001	< .001	< .001	< .001	< .001	< .001	< .001	< .001
	CAD4 vs CT	< .001	< .001	< .001	< .001	< .001	< .001	< .001	< .001	< .001
Sensitivity	CAD1 vs VR	1.000	0.462	1.000	1.000	1.000	1.000	1.000	1.000	1.000
	CAD2 vs VR	< .001	< .001	< .001	< .001	< .001	< .001	< .001	< .001	< .001
	CAD3 vs VR	1.000	0.692	0.721	1.000	1.000	1.000	1.000	1.000	1.000
	CAD4 vs VR	0.924	0.367	0.413	0.617	0.531	0.425	0.462	0.823	0.092
	CT vs VR	< .001	< .001	< .001	< .001	< .001	< .001	< .001	< .001	< .001
	CAD1 vs CT	1.000	1.000	0.109	0.321	< .001	0.260	0.533	1.000	0.839
	CAD2 vs CT	0.685	< .001	< .001	< .001	< .001	< .001	< .001	< .001	< .001
	CAD3 vs CT	0.685	< .001	< .001	< .001	1.000	0.135	0.043	< .001	< .001
	CAD4 vs CT	0.065	1.000	< .001	1.000	0.353	0.397	0.993	0.983	1.000
Specificity	CAD1 vs VR	0.075	< .001	< .001	0.113	< .001	< .001	< .001	< .001	< .001
	CAD2 vs VR	0.228	1.000	1.000	1.000	1.000	1.000	1.000	1.000	1.000
	CAD3 vs VR	0.195	0.713	< .001	1.000	< .001	< .001	0.151	1.000	0.063
	CAD4 vs VR	< .001	< .001	0.210	< .001	< .001	< .001	< .001	< .001	< .001
	CT vs VR	0.052	< .001	< .001	< .001	< .001	< .001	< .001	< .001	< .001
	CAD1 vs CT	0.133	0.063	0.248	< .001	1.000	0.087	0.131	0.633	0.133
	CAD2 vs CT	0.957	0.084	< .001	0.631	< .001	< .001	0.079	1.000	0.102
	CAD3 vs CT	0.092	< .001	< .001	< .001	< .001	< .001	< .001	< .001	< .001
	CAD4 vs CT	1.000	< .001	< .001	0.076	< .001	< .001	< .001	0.203	< .001
Accuracy	CAD1 vs VR	0.725	0.073	< .001	0.813	< .001	< .001	0.005	0.054	< .001
	CAD2 vs VR	< .001	0.006	0.702	< .001	< .001	0.079	0.013	< .001	< .001
	CAD3 vs VR	0.833	1.000	0.073	1.000	0.081	0.225	0.877	0.999	0.899
	CAD4 vs VR	0.002	0.452	1.000	0.266	0.169	0.102	0.768	0.157	0.432
	CT vs VR	< .001	< .001	< .001	< .001	< .001	< .001	< .001	< .001	< .001

Note. Group comparisons are performed using Chi-Square Test; 2-sided $p<0.05$ was considered statistically significant following Bonferroni correction for multiple comparisons. SDCT=standard dose computed tomography, LDCT=low dose Computed tomography, ULDCT=ultra low dose computed tomography, VR = volume rendering.

Table S3. Comparison of diagnostic performance for manual reading.

Scanning parameter	Metric	SDCT						LDCT						ULDCT					
		Br40			Br64			Br40			Br64			Br40			Br64		
		FBP	A-3	A-5	FBP	A-3	A-5	FBP	A-3	A-5	FBP	A-3	A-5	FBP	A-3	A-5	FBP	A-3	A-5
ObD_CT VS ObA_VR	Sensitivity	0.050	0.273	0.651	0.000	0.003	0.020	0.000	0.005	0.001	0.000	0.010	0.012	0.002	0.004	0.011	0.000	0.000	0.001
ObD_CT VS ObB_VR		0.000	0.030	0.062	0.000	0.000	0.002	0.000	0.001	0.009	0.000	0.000	0.003	0.000	0.000	0.000	0.000	0.000	0.000
ObD_CT VS ObC_VR		0.000	0.030	0.062	0.000	0.000	0.002	0.000	0.001	0.009	0.000	0.000	0.003	0.000	0.000	0.000	0.000	0.000	0.000
ObD_CT VS ObD_VR		0.000	0.001	0.006	0.000	0.000	0.002	0.000	0.000	0.000	0.000	0.000	0.000	0.000	0.000	0.000	0.000	0.000	0.000
ObD_CT VS ObA_VR	Specificity	1.000	1.000	1.000	1.000	1.000	1.000	1.000	1.000	1.000	1.000	1.000	1.000	1.000	1.000	1.000	0.893	1.000	1.000
ObD_CT VS ObB_VR		1.000	1.000	1.000	1.000	1.000	1.000	1.000	1.000	1.000	1.000	1.000	1.000	1.000	1.000	1.000	0.153	1.000	1.000
ObD_CT VS ObC_VR		1.000	1.000	1.000	1.000	1.000	1.000	1.000	1.000	1.000	1.000	1.000	1.000	1.000	1.000	1.000	0.153	1.000	1.000
ObD_CT VS ObD_VR		1.000	1.000	1.000	1.000	1.000	1.000	1.000	1.000	1.000	1.000	1.000	1.000	1.000	1.000	1.000	0.045	1.000	1.000
ObD_CT VS ObA_VR	Accuracy	1.000	1.000	1.000	0.522	0.743	0.675	0.455	0.470	0.099	0.335	1.000	1.000	0.461	0.329	0.422	0.000	0.087	0.130
ObD_CT VS ObB_VR		0.119	0.162	0.299	0.008	0.119	0.066	0.004	0.157	0.099	0.014	0.077	0.157	0.048	0.012	0.021	0.000	0.004	0.024
ObD_CT VS ObC_VR		0.119	0.162	0.299	0.018	0.119	0.066	0.004	0.157	0.099	0.014	0.077	0.157	0.048	0.012	0.021	0.000	0.004	0.044
ObD_CT VS ObD_VR		0.022	0.066	0.114	0.018	0.054	0.023	0.000	0.003	0.020	0.002	0.006	0.040	0.004	0.003	0.002	0.000	0.001	0.001

Note. Group comparisons are performed using Chi-Square Test; 2-sided p<0.05 was considered statistically significant following Bonferroni correction for multiple comparisons. ObD_CT=obsever D use original CT for image interpretaiton, SDCT=standard dose computed tomography, LDCT=low dose Computed tomography, ULDCT=ultra low dose computed tomography, VR = volume rendering, A-3= ADMIRE at the strength level of 3,A-5= ADMIRE at the strength level of 5.

Table S4. Comparison of sensitivity of four DL-CAD systems and manual reading in nodule subgroups.

Methods	Size				Density			Lung-RADS			
	≤5mm	5-10mm	10-15mm	15-20mm	SN	GGN	PSN	2	3	4A	4B
CAD1 vs CT	< .001	< .001	< .001	< .001	< .001	< .001	< .001	< .001	< .001	< .001	< .001
CAD2 vs CT	< .001	1.000	< .001	< .001	< .001	< .001	< .001	1.000	< .001	1.000	0.064
CAD3 vs CT	< .001	< .001	< .001	< .001	< .001	< .001	< .001	< .001	< .001	< .001	< .001
CAD4 vs CT	< .001	< .001	< .001	< .001	< .001	< .001	< .001	< .001	< .001	< .001	< .001
CAD1 vs VR	< .001	1.000	1.000	1.000	0.916	0.896	1.000	0.867	1.000	1.000	1.000
CAD2 vs VR	< .001	< .001	< .001	< .001	< .001	< .001	< .001	< .001	< .001	< .001	< .001
CAD3 vs VR	< .001	1.000	1.000	1.000	1.000	1.000	1.000	1.000	1.000	1.000	1.000
CAD4 vs VR	< .001	1.000	1.000	1.000	0.631	1.000	1.000	1.000	1.000	1.000	1.000
CT vs VR	< .001	< .001	< .001	< .001	< .001	< .001	< .001	< .001	< .001	< .001	< .001

Note. Group comparisons are performed using Chi-Square Test; 2-sided P<0.05 was considered statistically significant following Bonferroni correction for multiple comparisons. Lung-RADS=Lung CT Screening Reporting and Data Systems, VR=volume rendering, SN = solid nodules, GGN = ground glass nodules, PSN = part solid nodules

Table S5. Reading Time

Dose	Method	Radiologist A	Radiologist B	Radiologist C	Radiologist D
	P	< 0.001	< 0.001	< 0.001	< 0.001
SDCT	CT	142.61 ± 13.5	133.09 ± 8.4	129.67 ± 7.24	100.94 ± 10.19
	VR	103.39 ± 9.25	97.39 ± 5.85	95.18 ± 8.36	87.45 ± 9.34
	P	< 0.001	< 0.001	< 0.001	< 0.001
LDCT	CT	144.55 ± 12.76	134.76 ± 8.49	131.64 ± 7.7	103.67 ± 10.21
	VR	105.79 ± 9.39	102.27 ± 5.81	97.7 ± 8.11	89.39 ± 8.6
	P	< 0.001	< 0.001	< 0.001	< 0.001
ULDCT	CT	156.61 ± 15.49	152.48 ± 23.2	149.52 ± 26.38	141.58 ± 15.26
	VR	142.33 ± 14.32	131.67 ± 13.42	130.88 ± 14.03	122.03 ± 11.81

Comparison of reading time between reading method groups was performed with the Friedman test followed by the post-hoc test. 2-sided $P<0.05$ was considered statistically significant following Bonferroni correction for multiple comparisons. VR=volume rendering SDCT = standard dose computed tomography LDCT = low dose computed tomography ULDCT = ultra low dose computed tomography

Table S6. Comparison of Absolute Volume Error of four DL-CAD systems and manual reading.

Group comparison	Dose			Kernel		Algorithm			Total
	SDCT	LDCT	ULDCT	Br40	Br64	FBP	ADMIRE-3	ADMIRE-5	
CAD1 vs CT	< .001	< .001	< .001	< .001	< .001	< .001	< .001	< .001	< .001
CAD2 vs CT	1.000	1.000	< .001	< .001	< .001	< .001	1.000	< .001	< .001
CAD3 vs CT	1.000	< .001	< .001	< .001	< .001	< .001	< .001	< .001	< .001
CAD4 vs CT	< .001	< .001	< .001	0.134	< .001	< .001	< .001	< .001	< .001
CAD1 vs VR	< .001	< .001	< .001	< .001	< .001	< .001	< .001	< .001	< .001
CAD2 vs VR	1.000	1.000	1.000	0.335	1.000	1.000	0.156	1.000	0.015
CAD3 vs VR	1.000	< .001	0.056	< .001	1.000	1.000	0.022	0.034	< .001
CAD4 vs VR	0.017	< .001	< .001	< .001	< .001	< .001	< .001	< .001	< .001
CT vs VR	1.000	< .001	0.096	0.002	1.000	1.000	1.000	0.156	1.000

Comparison of AVE between groups was performed with repeated measures analysis of variance with the Green House-Geisser correction followed by post hoc test. 2-sided $P<0.05$ was considered statistically significant following Bonferroni correction for multiple comparisons. VR = volume rendering, SDCT = standard dose computed tomography, LDCT = low dose computed tomography, ULDCT = ultra low dose computed tomography.

Table S7. Subgroup Analysis of Absolute Volume Error

		Size (%)				Density (%)			Lung RADS (%)			
		<5mm	5-10mm	10-15mm	15-20mm	SN	GGN	PSN	2	3	4A	4B
CT		**22.47**	**21.16***	**12.35**	**5.63**	**15.80**	**19.94**	**6.75**	**20.72**	**7.40**	**12.97**	**5.99**
	Standard	20.28	18.26	9.36	4.09	15.78	13.37	5.05	15.68	5.30	13.33	5.82
	Low	20.41	19.61	10.17	6.14	12.48	19.58	6.89	19.77	7.44	9.77	4.08
	Ultralow	26.72	25.61	17.52	6.66	19.15	26.86	8.31	26.69	9.46	15.82	8.06
VR		**22.51**	**16.21**	**12.93**	**8.96**	**14.21**	**18.96**	**9.40**	**17.43**	**8.61**	**14.71**	**8.95**
	Standard	16.13	10.39	8.08	6.09	9.60	12.11	6.26	11.48	5.98	9.68	5.90
	Low	21.92	15.15	14.84	10.54	13.73	20.17	10.96	18.89	9.73	12.94	11.16
	Ultralow	25.47	23.09	15.87	10.26	19.29	24.62	10.97	21.91	10.13	21.51	9.78
CAD1		**28.91**	**31.70***	**24.10***	**56.16***	**27.24***	**24.23**	**55.06***	**26.02**	**44.64***	**35.64***	**47.89***
	Standard	31.07	31.92#	26.65#	72.09#	28.50#	24.38	71.30#	26.67#	62.52#	38.87#	52.40#
	Low	29.20	31.95#	23.49	54.47#	27.35#	24.94	52.86#	26.61	41.39#	35.27#	47.40#
	Ultralow	26.47	31.23	22.17	41.93#	25.89	23.36	41.03#	24.78	30.02	32.78	43.88#
CAD2		**31.69**	**13.46**	**8.73**	**11.73**	**12.01**	**18.64**	**11.74**	**23.14**	**9.23**	**7.09**	**9.46**
	Standard	34.90#	15.53	10.83	10.62	13.58	23.32	11.55	27.40#	9.88	7.30	10.71
	Low	31.09	14.29	7.93	10.83	11.54	18.99	11.04	22.81	9.10	6.47	9.24
	Ultralow	29.10	10.54	7.42	13.74	10.93	13.60	12.65	19.21	8.71	7.49	8.44
CAD3		**18.79**	**8.91**	**6.58**	**9.10**	**11.83**	**6.57***	**9.39**	**10.82**	**8.51**	**9.32**	**6.80**
	Standard	23.11	12.46	8.86	15.26	16.39	7.63	15.77	12.51	14.59	14.53	11.37
	Low	16.77	6.73	5.09	2.76	9.97	5.22#	3.53	8.97	3.77	6.35	2.85
	Ultralow	16.51	7.54	5.78	9.28	9.12	6.85#	8.87	10.98	7.18	7.08	6.18
CAD4		**29.05**	**30.02***	**20.97***	**45.36***	**24.65***	**24.96**	**43.91***	**26.25**	**34.06***	**29.72***	**41.02***
	Standard	31.49	27.54#	17.48	39.68#	21.12	26.83#	37.85#	28.07#	30.77#	23.19	31.78#
	Low	29.20	31.95#	23.49	54.47#	27.35#	24.94	52.86#	26.15	41.39#	35.27#	47.40#
	Ultralow	26.47	30.56	21.93	41.93#	25.50	23.09	41.03#	24.52	30.02	30.72	43.72#

Note. Data are percentages presented as means. Group comparisons are performed with VR as reference with repeated measures analysis of variance with the Green House-Geisser correction followed by post hoc test. 2-sided $p<.05$ was considered statistically significant following Bonferroni correction for multiple comparisons.*significant difference was observed; #significant difference was observed at the same scanning dose. AVE = Absolute Volume Error, Lung-RADS=Lung CT Screening Reporting and Data Systems, VR= volume rendering, SN = solid nodules, GGN = ground glass nodules, PSN = part solid nodule.

Table S8. Logistic Regression Analysis of Influencing Factors Associated with Detection Rates

	CT		VR		CAD1		CAD2		CAD3		CAD4	
	Odds Rate	p	Odds Rate	p	Odds Rate	p	Odds Rate	p	Odds Rate	p	Odds Rate	p
**Nodule density, n(%)**												
SN	Reference,1		Reference,1		Reference,1		Reference,1		Reference,1		Reference,1	
GGN	0.61 (0.48 ~ 0.77)	**<.001**	0.84 (0.77 ~ 0.92)	**<.001**	0.47 (0.36 ~ 0.61)	**<.001**	0.37 (0.27 ~ 0.51)	**<.001**	0.38 (0.28 ~ 0.50)	**<.001**	0.28 (0.22 ~ 0.37)	**<.001**
PSN	0.47 (0.28 ~ 0.79)	**0.005**	4.13 (3.55 ~ 4.80)	**<.001**	0.33 (0.24 ~ 0.46)	**<.001**	0.14 (0.10 ~ 0.21)	**<.001**	0.15 (0.11 ~ 0.21)	**<.001**	0.65 (0.47 ~ 0.91)	**0.013**
**Diameter, n(%)**												
0-5mm	Reference,1		Reference,1		Reference,1		Reference,1		Reference,1		Reference,1	
5-10mm	3.49 (2.73 ~ 4.45)	**<.001**	4.67 (4.17 ~ 5.24)	**<.001**	5.66 (4.37 ~ 7.32)	**<.001**	7.79 (5.68 ~ 10.67)	**<.001**	9.01 (6.79 ~ 11.95)	**<.001**	6.33 (4.85 ~ 8.26)	**<.001**
10-15mm	12.50 (9.81 ~ 15.92)	**<.001**	8.68 (7.68 ~ 9.80)	**<.001**	12.42 (9.65 ~ 15.99)	**<.001**	28.90 (21.16 ~ 39.46)	**<.001**	20.85 (15.79 ~ 27.54)	**<.001**	16.46 (12.66 ~ 21.39)	**<.001**
15-20mm	30.23 (19.07 ~ 47.92)	**<.001**	18.38 (15.26 ~ 22.14)	**<.001**	23.57 (16.57 ~ 33.53)	**<.001**	47.48 (31.55 ~ 71.47)	**<.001**	45.51 (31.45 ~ 65.86)	**<.001**	17.18 (11.98 ~ 24.64)	**<.001**
**Location, n(%)**												
Peripheral	Reference,1		Reference,1		Reference,1		Reference,1		Reference,1		Reference,1	
Central	0.96 (0.87 ~ 1.07)	0.454	1.29 (1.18 ~ 1.40)	**<.001**	0.85 (0.78 ~ 0.92)	**<.001**	1.11 (1.01 ~ 1.22)	**0.029**	0.90 (0.82 ~ 0.98)	**0.018**	0.99 (0.91 ~ 1.09)	0.902
**LungRADS, n(%)**												
2	Reference,1		Reference,1		Reference,1		Reference,1		Reference,1		Reference,1	
3	2.63 (1.59 ~ 4.35)	**<.001**	6.69 (5.61 ~ 7.98)	**<.001**	1.49 (1.03 ~ 2.15)	**0.033**	3.17 (2.07 ~ 4.87)	**<.001**	1.90 (1.30 ~ 2.79)	**0.001**	0.50 (0.35 ~ 0.74)	**<.001**
4A	3.54 (2.67 ~ 4.71)	**<.001**	5.92 (5.23 ~ 6.72)	**<.001**	1.18 (0.89 ~ 1.56)	0.262	2.98 (2.11 ~ 4.22)	**<.001**	1.21 (0.88 ~ 1.64)	0.236	0.94 (0.70 ~ 1.26)	0.679
4B	1.04 (0.71 ~ 1.53)	0.828	4.67 (3.73 ~ 5.86)	**<.001**	0.85 (0.60 ~ 1.19)	0.331	0.92 (0.62 ~ 1.37)	0.687	0.77 (0.54 ~ 1.11)	0.16	0.38 (0.27 ~ 0.54)	**<.001**
**Dose, n(%)**												
Standard	Reference,1		Reference,1		Reference,1		Reference,1		Reference,1		Reference,1	
Low	0.47 (0.41 ~ 0.54)	**<.001**	0.58 (0.52 ~ 0.65)	**<.001**	0.69 (0.62 ~ 0.77)	**<.001**	0.63 (0.56 ~ 0.70)	**<.001**	0.65 (0.58 ~ 0.72)	**<.001**	0.63 (0.57 ~ 0.71)	**<.001**
Ultra-low	0.24 (0.21 ~ 0.27)	**<.001**	0.37 (0.33 ~ 0.41)	**<.001**	0.44 (0.39 ~ 0.49)	**<.001**	0.37 (0.33 ~ 0.42)	**<.001**	0.34 (0.31 ~ 0.38)	**<.001**	0.36 (0.32 ~ 0.40)	**<.001**
**Kernel, n(%)**												
Br40	Reference,1		Reference,1		Reference,1		Reference,1		Reference,1		Reference,1	
Br64	0.53 (0.47 ~ 0.58)	**<.001**	0.66 (0.60 ~ 0.71)	**<.001**	0.78 (0.72 ~ 0.85)	**<.001**	0.50 (0.46 ~ 0.55)	**<.001**	0.49 (0.45 ~ 0.53)	**<.001**	0.61 (0.56 ~ 0.66)	**<.001**
**Algorithm, n(%)**												
FBP	Reference,1		Reference,1		Reference,1		Reference,1		Reference,1		Reference,1	
ADMIRE-3	1.65 (1.46 ~ 1.85)	**<.001**	1.41 (1.27 ~ 1.55)	**<.001**	1.46 (1.32 ~ 1.62)	**<.001**	1.61 (1.44 ~ 1.80)	**<.001**	1.74 (1.56 ~ 1.93)	**<.001**	1.54 (1.39 ~ 1.71)	**<.001**
ADMIRE-5	3.08 (2.71 ~ 3.50)	**<.001**	2.17 (1.95 ~ 2.42)	**<.001**	2.10 (1.89 ~ 2.33)	**<.001**	2.48 (2.21 ~ 2.79)	**<.001**	2.88 (2.58 ~ 3.21)	**<.001**	2.30 (2.06 ~ 2.56)	**<.001**

Note. Data in parentheses are 95% CIs. Bold text are found statistically significant. VR = volume rendering SDCT=standard dose computed tomography LDCT=low dose Computed tomography ULDCT=ultra low dose computed tomography Lung-RADS=Lung CT Screening Reporting and Data Systems SN = solid nodules GGN = ground glass nodules PSN = part solid nodules

Table S9. Multivariable Generalized Linear Mixed Model for Influencing Factors for AVE

	CT		VR		CAD1		CAD2		CAD3		CAD4	
	β	P	*β*	P	*β*	P	*β*	P	*β*	P	*β*	P
**Nodule density, n(%)**												
SN	Reference,0		Reference,0		Reference,0		Reference,0		Reference,0		Reference,0	
GGN	-0.21 (-0.29 ~ -0.14)	**<.001**	-0.22 (-0.37 ~ -0.07)	**0.004**	-0.11 (-0.16 ~ -0.07)	**<.001**	-0.16 (-0.20 ~ -0.13)	**<.001**	-0.15 (-0.18 ~ -0.12)	**<.001**	-0.13 (-0.18 ~ -0.09)	**<.001**
PSN	0.05 (-0.01 ~ 0.11)	0.093	0.05 (-0.07 ~ 0.18)	0.408	0.66 (0.61 ~ 0.72)	**<.001**	0.11 (0.07 ~ 0.16)	**<.001**	0.07 (0.04 ~ 0.11)	**<.001**	0.52 (0.46 ~ 0.57)	**<.001**
**Diameter, n(%)**												
0-5mm	Reference,0		Reference,0		Reference,0		Reference,0		Reference,0		Reference,0	
5-10mm	0.20 (0.13 ~ 0.28)	**<.001**	0.16 (0.01 ~ 0.31)	**0.037**	0.14 (0.10 ~ 0.19)	<.001	0.11 (0.07 ~ 0.15)	**<.001**	0.05 (0.02 ~ 0.08)	**0.001**	0.16 (0.11 ~ 0.21)	**<.001**
10-15mm	0.12 (0.05 ~ 0.19)	**<.001**	0.10 (-0.05 ~ 0.24)	0.197	0.03 (-0.02 ~ 0.07)	**0.241**	0.06 (0.03 ~ 0.10)	**<.001**	0.03 (0.01 ~ 0.06)	**0.023**	0.05 (0.01 ~ 0.10)	**0.024**
15-20mm	0.09 (0.01 ~ 0.18)	**0.037**	0.08 (-0.10 ~ 0.25)	0.39	-0.09 (-0.15 ~ -0.02)	**0.008**	0.04 (-0.01 ~ 0.09)	0.086	0.00 (-0.04 ~ 0.04)	0.889	-0.03 (-0.09 ~ 0.04)	0.408
**Location, n(%)**												
Peripheral	Reference,0		Reference,0		Reference,0		Reference,0		Reference,0		Reference,0	
Central	-0.04 (-0.06 ~ -0.02)	**<.001**	-0.03 (-0.07 ~ 0.00)	0.086	-0.00 (-0.02 ~ 0.01)	0.662	-0.00 (-0.02 ~ 0.01)	0.697	-0.01 (-0.02 ~ -0.01)	**0.013**	-0.00 (-0.02 ~ 0.01)	0.848
**LungRADS, n(%)**												
2	Reference,0		Reference,0		Reference,0		Reference,0		Reference,0		Reference,0	
3	-0.31 (-0.40 ~ -0.22)	**<.001**	-0.30 (-0.48 ~ -0.12)	**<.001**	-0.44 (-0.51 ~ -0.37)	**<.001**	-0.27 (-0.32 ~ -0.22)	**<.001**	-0.17 (-0.21 ~ -0.13)	**<.001**	-0.44 (-0.50 ~ -0.37)	**<.001**
4A	-0.33 (-0.40 ~ -0.25)	**<.001**	-0.32 (-0.47 ~ -0.16)	**<.001**	-0.10 (-0.16 ~ -0.05)	**<.001**	-0.23 (-0.27 ~ -0.19)	**<.001**	-0.13 (-0.16 ~ -0.10)	**<.001**	-0.14 (-0.20 ~ -0.09)	**<.001**
4B	-0.31 (-0.39 ~ -0.23)	**<.001**	-0.29 (-0.46 ~ -0.12)	**<.001**	-0.11 (-0.17 ~ -0.05)	**<.001**	-0.22 (-0.27 ~ -0.17)	**<.001**	-0.16 (-0.19 ~ -0.12)	**<.001**	-0.13 (-0.19 ~ -0.07)	**<.001**
**Dose, n(%)**												
Standard	Reference,0		Reference,0		Reference,0		Reference,0		Reference,0		Reference,0	
Low	0.01 (-0.01 ~ 0.03)	0.422	-0.02 (-0.06 ~ 0.02)	0.25	-0.05 (-0.07 ~ -0.03)	**<.001**	-0.01 (-0.02 ~ 0.01)	0.234	-0.06 (-0.07 ~ -0.05)	**<.001**	0.08 (0.07 ~ 0.10)	**<.001**
Ultra-low	0.00 (-0.02 ~ 0.02)	0.73	-0.01 (-0.05 ~ 0.03)	0.659	-0.10 (-0.12 ~ -0.08)	**<.001**	-0.01 (-0.02 ~ 0.01)	0.232	-0.06 (-0.07 ~ -0.04)	**<.001**	0.02 (0.01 ~ 0.04)	**0.022**
**Kernel, n(%)**												
Br40	Reference,0		Reference,0		Reference,0		Reference,0		Reference,0		Reference,0	
Br64	-0.03 (-0.04 ~ -0.01)	**0.002**	-0.03 (-0.06 ~ 0.00)	0.072	-0.01 (-0.03 ~ 0.00)	**0.112**	-0.03 (-0.05 ~ -0.02)	**<.001**	0.02 (0.01 ~ 0.03)	**<.001**	0.05 (0.04 ~ 0.07)	**<.001**
**Algorithm, n(%)**												
FBP	Reference,0		Reference,0		Reference,0		Reference,0		Reference,0		Reference,0	
ADMIRE-3	0.03 (0.01 ~ 0.05)	**0.008**	-0.02 (-0.06 ~ 0.02)	0.302	0.01 (-0.01 ~ 0.03)	0.24	-0.01 (-0.02 ~ 0.01)	0.464	-0.07 (-0.08 ~ -0.06)	**<.001**	-0.01 (-0.03 ~ 0.01)	0.425
ADMIRE-5	-0.02 (-0.04 ~ -0.01)	**0.027**	-0.04 (-0.08 ~ 0.00)	0.053	0.04 (0.02 ~ 0.06)	<.001	0.00 (-0.01 ~ 0.02)	0.916	-0.07 (-0.08 ~ -0.06)	**<.001**	0.03 (0.01 ~ 0.05)	**0.006**

Note. Data in parentheses are 95% CIs. Bold text are found statistically significant. AVE = absolute volume error VR = volume rendering SDCT=standard dose computed tomography LDCT=low dose Computed tomography ULDCT=ultra low dose computed tomography Lung-RADS=Lung CT Screening Reporting and Data Systems SN = solid nodules GGN = ground glass nodules PSN = part solid nodules

Table S10. Performance of nodule density type classification of three automatic DL-CAD

Method	Dose	SN%	GGN%	PSN%	Total%
CAD1	**Total**	**35.15**	**42.35**	**0.89**	**28.31**
	Standard	45.10	33.64	0.44	30.44
	Low	39.24	43.76	0.88	31.32
	Ultralow	21.10	49.65	1.36	23.17
CAD3	**Total**	**10.29**	**27.20**	**2.92**	**12.60**
	Standard	17.28	16.20	1.75	13.12
	Low	9.75	20.63	3.51	11.65
	Ultralow	3.82	44.75	3.51	13.02
CAD4	**Total**	**0.16**	**27.60**	**41.82**	**18.32**
	Standard	0.00	24.86	45.61	19.17
	Low	0.00	22.86	40.35	17.09
	Ultralow	0.49	35.10	39.50	18.69

Note. Data are percentages presented as means.SN = solid nodules, GGN = ground glass nodules, PSN = part solid nodules

Table S11. Logistic Regression Analysis of Influencing Factors Associated with Lung-RADS Classification

	CT		VR		CAD1		CAD2		CAD3		CAD4	
	Odds Rate	p	Odds Rate	p	Odds Rate	p	Odds Rate	p	Odds Rate	p	Odds Rate	p
**Nodule density, n(%)**												
SN	Reference,1		Reference,1		Reference,1		Reference,1		Reference,1		Reference,1	
GGN	0.61 (0.48 ~ 0.77)	**<.001**	0.84 (0.77 ~ 0.92)	**<.001**	0.47 (0.36 ~ 0.61)	**<.001**	0.37 (0.27 ~ 0.51)	**<.001**	0.38 (0.28 ~ 0.50)	**<.001**	0.28 (0.22 ~ 0.37)	**<.001**
PSN	0.47 (0.28 ~ 0.79)	**0.005**	4.13 (3.55 ~ 4.80)	**<.001**	0.33 (0.24 ~ 0.46)	**<.001**	0.14 (0.10 ~ 0.21)	**<.001**	0.15 (0.11 ~ 0.21)	**<.001**	0.65 (0.47 ~ 0.91)	**0.013**
**Diameter, n(%)**												
0-5mm	Reference,1		Reference,1		Reference,1		Reference,1		Reference,1		Reference,1	
5-10mm	3.49 (2.73 ~ 4.45)	**<.001**	4.67 (4.17 ~ 5.24)	**<.001**	5.66 (4.37 ~ 7.32)	**<.001**	7.79 (5.68 ~ 10.67)	**<.001**	9.01 (6.79 ~ 11.95)	**<.001**	6.33 (4.85 ~ 8.26)	**<.001**
10-15mm	12.50 (9.81 ~ 15.92)	**<.001**	8.68 (7.68 ~ 9.80)	**<.001**	12.42 (9.65 ~ 15.99)	**<.001**	28.90 (21.16 ~ 39.46)	**<.001**	20.85 (15.79 ~ 27.54)	**<.001**	16.46 (12.66 ~ 21.39)	**<.001**
15-20mm	30.23 (19.07 ~ 47.92)	**<.001**	18.38 (15.26 ~ 22.14)	**<.001**	23.57 (16.57 ~ 33.53)	**<.001**	47.48 (31.55 ~ 71.47)	**<.001**	45.51 (31.45 ~ 65.86)	**<.001**	17.18 (11.98 ~ 24.64)	**<.001**
**Location, n(%)**												
Peripheral	Reference,1		Reference,1		Reference,1		Reference,1		Reference,1		Reference,1	
Central	0.96 (0.87 ~ 1.07)	0.454	1.29 (1.18 ~ 1.40)	**<.001**	0.85 (0.78 ~ 0.92)	**<.001**	1.11 (1.01 ~ 1.22)	**0.029**	0.90 (0.82 ~ 0.98)	**0.018**	0.99 (0.91 ~ 1.09)	0.902
**LungRADS, n(%)**												
2	Reference,1		Reference,1		Reference,1		Reference,1		Reference,1		Reference,1	
3	2.63 (1.59 ~ 4.35)	**<.001**	6.69 (5.61 ~ 7.98)	**<.001**	1.49 (1.03 ~ 2.15)	**0.033**	3.17 (2.07 ~ 4.87)	**<.001**	1.90 (1.30 ~ 2.79)	**0.001**	0.50 (0.35 ~ 0.74)	**<.001**
4A	3.54 (2.67 ~ 4.71)	**<.001**	5.92 (5.23 ~ 6.72)	**<.001**	1.18 (0.89 ~ 1.56)	0.262	2.98 (2.11 ~ 4.22)	**<.001**	1.21 (0.88 ~ 1.64)	0.236	0.94 (0.70 ~ 1.26)	0.679
4B	1.04 (0.71 ~ 1.53)	0.828	4.67 (3.73 ~ 5.86)	**<.001**	0.85 (0.60 ~ 1.19)	0.331	0.92 (0.62 ~ 1.37)	0.687	0.77 (0.54 ~ 1.11)	0.16	0.38 (0.27 ~ 0.54)	**<.001**
**Dose, n(%)**												
Standard	Reference,1		Reference,1		Reference,1		Reference,1		Reference,1		Reference,1	
Low	0.47 (0.41 ~ 0.54)	**<.001**	0.58 (0.52 ~ 0.65)	**<.001**	0.69 (0.62 ~ 0.77)	**<.001**	0.63 (0.56 ~ 0.70)	**<.001**	0.65 (0.58 ~ 0.72)	**<.001**	0.63 (0.57 ~ 0.71)	**<.001**
Ultra-low	0.24 (0.21 ~ 0.27)	**<.001**	0.37 (0.33 ~ 0.41)	**<.001**	0.44 (0.39 ~ 0.49)	**<.001**	0.37 (0.33 ~ 0.42)	**<.001**	0.34 (0.31 ~ 0.38)	**<.001**	0.36 (0.32 ~ 0.40)	**<.001**
**Kernel, n(%)**												
Br40	Reference,1		Reference,1		Reference,1		Reference,1		Reference,1		Reference,1	
Br64	0.53 (0.47 ~ 0.58)	**<.001**	0.66 (0.60 ~ 0.71)	**<.001**	0.78 (0.72 ~ 0.85)	**<.001**	0.50 (0.46 ~ 0.55)	**<.001**	0.49 (0.45 ~ 0.53)	**<.001**	0.61 (0.56 ~ 0.66)	**<.001**
**Algorithm, n(%)**												
FBP	Reference,1		Reference,1		Reference,1		Reference,1		Reference,1		Reference,1	
ADMIRE-3	1.65 (1.46 ~ 1.85)	**<.001**	1.41 (1.27 ~ 1.55)	**<.001**	1.46 (1.32 ~ 1.62)	**<.001**	1.61 (1.44 ~ 1.80)	**<.001**	1.74 (1.56 ~ 1.93)	**<.001**	1.54 (1.39 ~ 1.71)	**<.001**
ADMIRE-5	3.08 (2.71 ~ 3.50)	**<.001**	2.17 (1.95 ~ 2.42)	**<.001**	2.10 (1.89 ~ 2.33)	**<.001**	2.48 (2.21 ~ 2.79)	**<.001**	2.88 (2.58 ~ 3.21)	**<.001**	2.30 (2.06 ~ 2.56)	**<.001**

Note. Data in parentheses are 95% CIs. Bold text are found statistically significant. VR = thin slab volume rendering SDCT=standard dose computed tomography LDCT=low dose Computed tomography ULDCT=ultra low dose computed tomography Lung-RADS=Lung CT Screening Reporting and Data Systems SN = solid nodules GGN = ground glass nodules PSN = part solid nodules

Table S12. Literature summary of commercial DL-CAD systems for pulmonary nodule detection during the past five years (2021-2025)

doi	DL-CAD	Models	Data	Performance	Pros	Cons
https://doi.org/10.1016/j.crad.2021.07.012	AVIEW LCS	DenseNet and ResNetXt	6267 real nodules	Sensitivity:88%	Big datasets and validation the pathology	Lack of different scanning doses and only a standalone DL-CAD system
10.1007/s00330-023-09525-z	InferRead CT Lung, InferVision Medical Health	DenseNet and Faster-R-net	326 simulated nodules	Sensitivity:97.9%	Different scanning doses and reconstructions	only a standalone DL-CAD system
https://doi.org/10.1016/j.diii.2021.12.002	InferRead CT Lung, InferVision Medical Health	DenseNet and Faster-R-net	75 simulated nodules	Sensitivity:30%-100%	Different scanning doses and reconstructions	only a standalone DL-CAD system
https://doi.org/10.1016/j.compbiomed.2022.105538	InferRead CT Lung, InferVision Medical Health	DenseNet and Faster-R-net	1310 real nodules	Sensitivity: 70.15%-78.03%	Different scanning doses and reconstructions	only a standalone DL-CAD system
https://doi.org/10.1186/s12880-024-01288-3	InferRead CT Lung, InferVision Medical Health	DenseNet and Faster-R-net	570 real nodules	Reach AUC=0.921(0.884-0.949)	Validate the value of DL-CAD evaluate nodules' characterise	Lack of different scanning doses and only a standalone DL-CAD system
10.1002/acm2.13589	InferRead CT Lung, InferVision Medical Health	DenseNet and Faster-R-net	234 real nodules	Sensitivity of 95.8%, specificity of 77.8%	Evaluate the influence of different weight	only a standalone DL-CAD system
https://doi.org/10.1007/s11604-020-01009-0	InferRead CT Lung, InferVision Medical Health	DenseNet and Faster-R-net	1486 real nodules	Sensitivity of 70.3%	Validate the value of ultra low dose CT	Lack of different scanning doses and only a standalone DL-CAD system
https://doi.org/10.3390/diagnostics14222558	VUNO Med-LungCT AI	Unknown	533 real nodules	Reach AUC=0.891-0.921	Compare different slice thickness	Lack of different scanning doses and only a standalone DL-CAD system
https://doi.org/10.1148/radiol.2021203387	VUNO Med-LungCT AI	Unknown	424 real nodules	Sensitivity: 86.8%-94.1%	Compare different slice thickness	Lack of different scanning doses and only a standalone DL-CAD system
10.1007/s00330-021-08202-3	VUNO Med-LungCT AI	Unknown	200 real nodules	The consistency of DL-CAD in diagnosing Lung-RADS is 0.65	Evaluation of the Value of DL-CAD in Lung Nodule LungRADS Classification	Lack of different scanning doses and only a standalone DL-CAD system
10.21037/jtd-24-1311	The Lung Nodule Intelligent Auxiliary Analysis System,Shukun Technology	Modified FNP, UNet, Resnet	4319 real nodules	Accuracy: 97.87%-99.36%	Validate the clinical value of 1,024-matrix	Lack of different scanning doses and only a standalone DL-CAD system
https://doi.org/10.1186/s40644-024-00770-z	YITU AI	Resnet and CNN	240 simulated nodules	Sensitivity: 80%	Different scanning doses and reconstructions	only a standalone DL-CAD system
10.1186/s12880-025-01746-6	YITU AI	Resnet and CNN	535 real nodules	Sensitivity: 83.4%	Evaluate the value of deep learning reconstruction	Lack of different scanning doses and only a standalone DL-CAD system
10.1038/s41591-024-03211-3	uAI, United Imaging Healthcare	Cascade FPN and VB-Net	45064 real nodules	Reach AUC=0.918	Big datasets, validation the pathology and propose a Chinese Lung Nodules Reporting and Data System	Lack of different scanning doses and only a standalone DL-CAD system
https://doi.org/10.1148/ryai.2019180084	InferRead CT Lung, InferVision	Cascade FPN	58078 real	Reach AUC=0.953	Big datasets	Lack of different scanning

	Medical Health	and VB-Net	nodules			doses and only a standalone DL-CAD system
https://doi.org/10.1016/j.acra.2024.01.010	uAI, United Imaging Healthcare	Cascade FPN and VB-Net	104 real nodules	Reach AUC=0.831-0.876	Evaluate the value of ultra low dose	only a standalone DL-CAD system
10.1097/RLI.0000000000000713	syngo.via Lung CAD Siemens Healthcare	3D CNN and CNN	2451 real nodules	Sensitivity of 90.7%, specificity of 37.7%	Compare deep learning CAD with machine learning CAD	Lack of different scanning doses and only a standalone DL-CAD system
https://doi.org/10.1186/s12916-021-01928-3	syngo.via Lung CAD Siemens Healthcare	3D CNN and CNN	56 real nodules	Sensitivity of 100%, specificity of 37.8%	Evaluate the value of prognosis	Lack of different scanning doses and only a standalone DL-CAD system
10.1097/RTI.0000000000000613	syngo.via Lung CAD Siemens Healthcare	3D CNN and CNN	103 real nodules	Sensitivity: 96.1%	Compare time consuming	Lack of different scanning doses and only a standalone DL-CAD system
https://doi.org/10.3349/ymj.2024.0050	syngo.via Lung CAD Siemens Healthcare	3D CNN and CNN	269 real nodules	Sensitivity: 84%	Evaluate the value in Coronary Artery Calcium-Scoring CT for Pulmonary Nodule Detection	Lack of different scanning doses and only a standalone DL-CAD system
10.1007/s00330-022-08584-y	syngo.via Lung CAD Siemens Healthcare	3D CNN and CNN	173 real nodules	Sensitivity: 66%-98%	Different scanning doses and weight	only a standalone DL-CAD system
https://doi.org/10.1007/s00330-024-10969-0	Qure.ai	Unknown	183 real nodules	Sensitivity: 81.9%	Validate the value of DL-CAD evaluate nodules’ characterise	Lack of different scanning doses and only a standalone DL-CAD system
https://doi.org/10.1016/j.acra.2024.11.042	Deepwise	Unknown	385 real nodules	Sensitivity: 79.4%-95.2%	Evaluate the value of deep learning reconstruction	Lack of different scanning doses and only a standalone DL-CAD system
https://doi.org/10.1016/j.crad.2024.04.008	LungDoc 5.7	unknown	710 real nodules	Sensitivity: 96.88.4%-96.93%	Evaluate the value of deep learning reconstruction	Lack of different scanning doses and only a standalone DL-CAD system
10.1371/journal.pone.0297390	LuCAS	unknown	72 real nodules	64.7%-88.2% correctly classification of Lung-RADS	Evaluate the value of deep learning reconstruction	only a standalone DL-CAD system
10.21037/QIMS-22-1297	VoxelCloud	FPN and Resnet	5638 real nodules	Sensitivity: 90.19%	Big datasets	Lack of different scanning doses and only a standalone DL-CAD system
10.1148/radiol.212182	LCP-CNN CAD	unknown	300 real nodules	Sensitivity: 81.7%	Big datasets and validation the pathology	Lack of different scanning doses and only a standalone DL-CAD system
10.1371/journal.pone.0266799	Veye Chest	unknown	470 real nodules	Sensitivity: 80.3%	Big datasets	Lack of different scanning doses and only a standalone DL-CAD system
https://doi.org/10.1016/j.crad.2021.07.012	Veye Chest	unknown	91 real nodules	Sensitivity: 88%	Evaluate the value of DL-CAD	Lack of different scanning doses and only a standalone DL-CAD system
https://doi.org/10.1016/j.ejrad.2021.109526	Veye Chest	unknown	5786 real	F2 Score: 0.73±0.053	Compare different reconstruction	only a standalone DL-CAD

			nodules			system
10.3389/fonc.2020.545862	σ -Discover	unknown	261 real nodules	Sensitivity: 94.7%	Compare DL-CAD with double reading by radiologist	Lack of different scanning doses and only a standalone DL-CAD system
https://doi.org/10.1016/j.ejrad.2020.108928	YITU AI,InferRead CT Lung, InferVision Medical Health,IMsight and 12Sigma		540 simulated nodules	Sensitivity: 60%-100%	Compare different reconstruction,various scanning doses and four DL-CAD systems	dealt with simulated nodules
10.1038/s41598-024-73435-3	ClearRead AI and syngo.via Lung CAD Siemens Healthcare		228 real nodules	Sensitivity: 67%-86%	Compare various DL-CAD systems	Lack of different scanning doses and only a standalone DL-CAD system
10.2214/AJR.24.31972	Flyer Scan and Medical Open Network for Artificial Intelligence	unknown	247 real nodules	Sensitivity: 62.3%-76.9%	Compare different scanning doses and applying conditions	Lack of different reconstruction
10.2214/AJR.23.30345	Flyer Scan and Medical Open Network for Artificial Intelligence	unknown	288 real nodules	Sensitivity: 53.1%-68.4%	Compare different applying conditions	Lack of different scanning doses

Supplementary Figure S1. Comparison of pulmonary nodule visualization using axial CT, maximum intensity projection (MIP, slab thickness of 10mm), and volume rendering (VR, slab thickness of 10mm) techniques under low dose computed tomography (LDCT), and ultra-low dose computed tomography (ULDCT) protocols. **Left column**: 8 mm pure ground-glass nodule (-630 HU) in the left upper lobe (LDCT, Br64 kernel, FBP reconstruction). (Upper) Axial CT image; (Lower) MIP image where vascular interference reduces nodule visibility; (Middle) VR image demonstrating enhanced nodule-background contrast (brown nodule vs. blue parenchyma). **Right column**: 15 mm part-solid nodule with 5mm solid component (-650 HU ground-glass component, 0 HU solid component) in the right upper lobe (ULDCT, Br64 kernel, ADMIRE-3 reconstruction). (Upper) Axial CT image; (Lower) MIP image exhibiting prominent noise compromising diagnostic quality; (Middle) VR image maintaining diagnostic capacity through effective noise suppression while preserving nodule visibility.

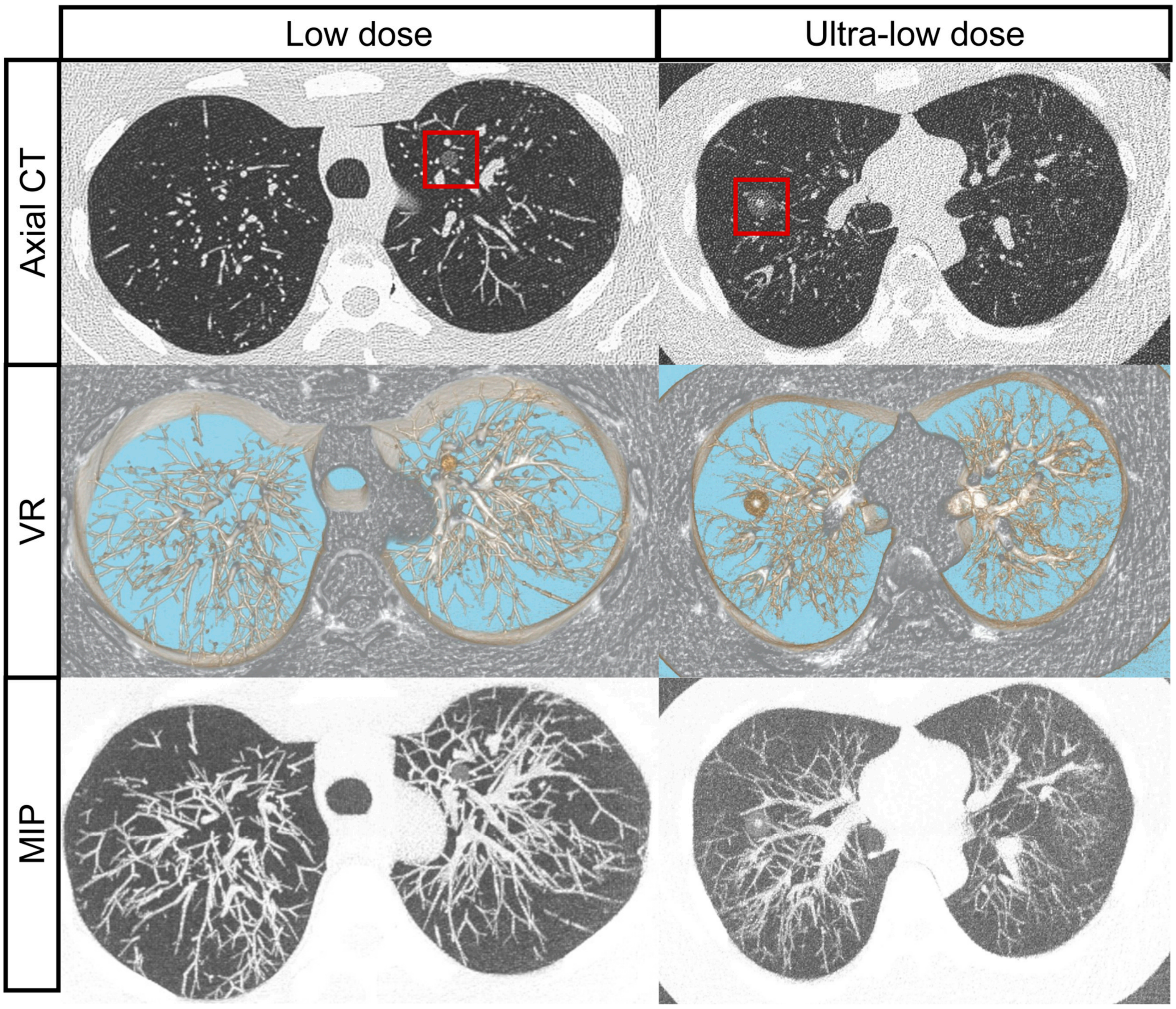

Supplementary Figure S2. Opacity-CT value curve setting of VR.

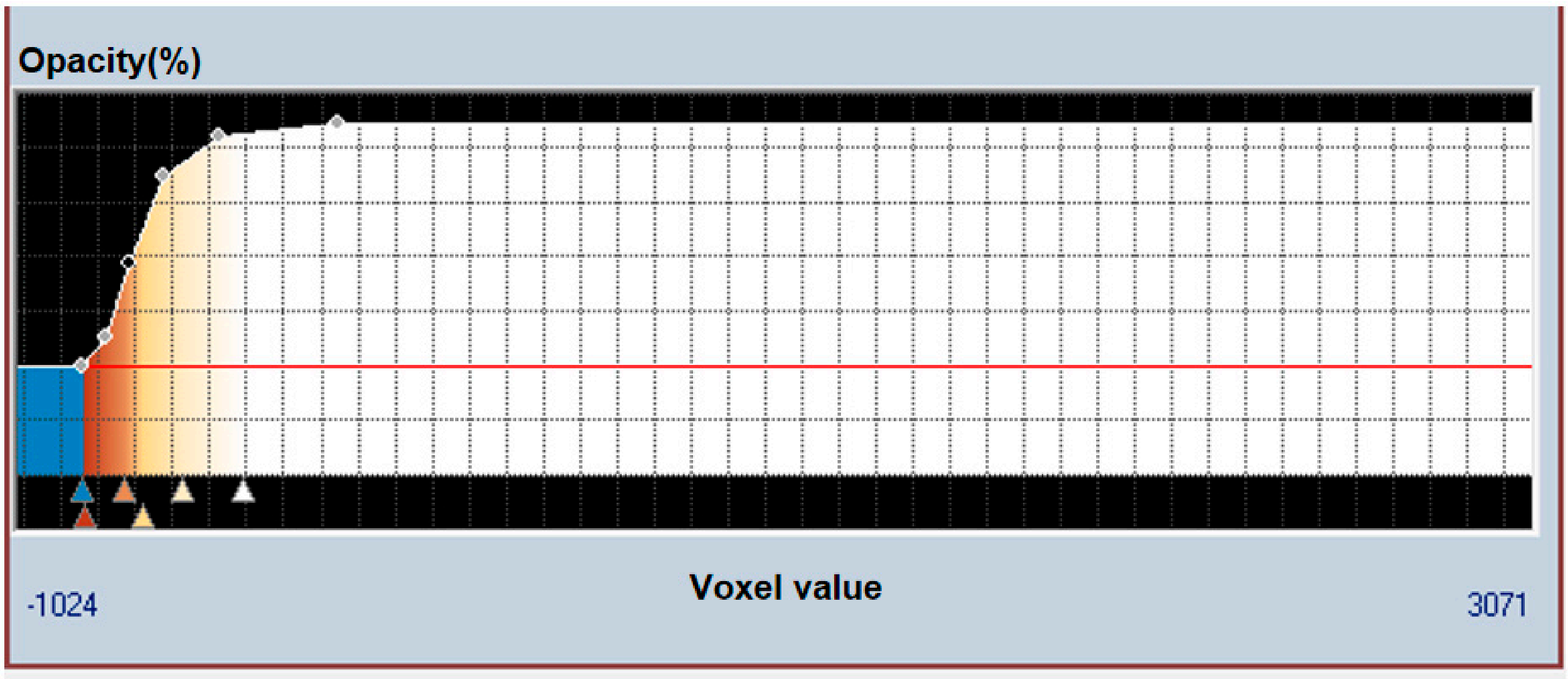

Supplementary Figure S3 shows subjective image quality ratings of two readers for the different scanning protocols. Subjective image quality was graded on a 5-point Likert scale: 1 = nondiagnostic image quality, strong artifacts, insufficient for diagnostic purposes score; 2 = severe artifacts with uncertainty about the evaluation; 3 = moderate artefacts with restricted assessment; 4 = slight artifacts with unrestricted diagnostic image evaluation possible; and 5 = excellent image quality, no artifacts. Image quality with a score of 3 to 5 on the Likert scale were considered diagnostic. *significant difference was observed.

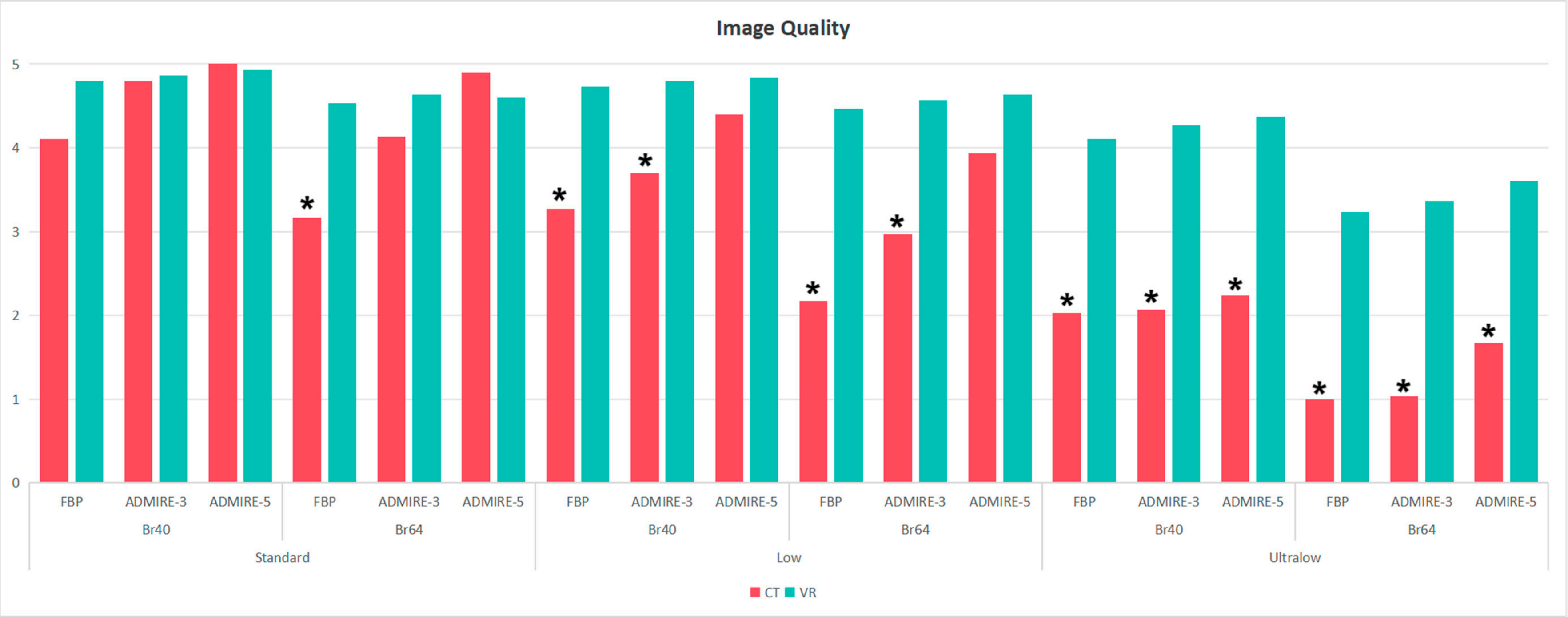

Supplement: Supplementary file 1 [file diagnostics-15-01623-s001.zip › diagnostics-3691310-SI.pdf]
